# Interleukin-6 Induced Proliferation Is Attenuated by Transforming Growth Factor-β-Induced Signaling in Human Hepatocellular Carcinoma Cells

**DOI:** 10.3389/fonc.2021.811941

**Published:** 2022-01-20

**Authors:** Abhilasha Srivastava, Harshita Sharma, Simran Khanna, Tejasvini Sadhu Balasundaram, Shibasish Chowdhury, Rajdeep Chowdhury, Sudeshna Mukherjee

**Affiliations:** Department of Biological Sciences, Birla Institute of Technology and Science (BITS) Pilani, Rajasthan, India

**Keywords:** IL-6, TGF-β, proliferation, EMT, p65

## Abstract

Hepatocellular carcinoma (HCC) is often associated with an inflammatory setting. A plethora of cytokines are secreted in this milieu, actively contributing to the progression of the disease; however, the extent of cytokine interaction and how it contributes to HCC development remains an enigma. In this regard, our analysis of available patient-derived data suggests that cytokines like interleukin-6 (IL-6) and transforming growth factor-beta (TGF-β) are enriched in HCC. We further analyzed the effect of these cytokines independently or in combination on HCC cells. Importantly, IL-6 was found to induce a STAT-3-dependent proliferation and mediate its pro-proliferative effects through activation and direct interaction with the p65 subunit of NFkB. Alternatively, TGF-β was found to induce a SMAD-dependent induction of epithelial to mesenchymal transition (EMT) coupled to growth arrest in these cells. Interestingly, the simultaneous addition of IL-6 and TGF-β failed to profoundly impact EMT markers but resulted in attenuation of IL-6-induced pro-proliferative effects. Analysis of the putative molecular mechanism revealed a decrease in IL-6 receptor (IL-6R) transcript levels, reduced expression of IL-6-induced STAT-3, and its nuclear localization upon addition of TGF-β along with IL-6. Consequently, a reduced p65 activation was also observed in combination treatment. Importantly, SMAD levels were unperturbed and the cells showed more TGF-β-like features under combination treatment. Finally, we observed that TGF-β resulted in enrichment of repressive chromatin mark (H3K27me3) coupled to growth arrest, while IL-6 induced an open chromatin signature (H3K4me3) associated with an enhanced expression of EZH2. Overall, for the first time, we show that TGF-β attenuates IL-6-induced effects by regulating the receptor level, downstream signaling, and the epigenome. Understanding the complex interactions between these cytokines can be imperative to a better understanding of the disease, and manipulation of cytokine balance can act as a prospective future therapeutic strategy.

## Introduction

Hepatocellular carcinoma (HCC) is one of the topmost cancers worldwide, with an increasingly high incidence rate and mortality (1). It is a very aggressive disease and is often associated with frequent malignancy. Consequently, patients suffering from HCC have poor survival expectancy. To date, surgery is the first-line treatment option for HCC; however, a high rate of intrahepatic metastases often renders patients ineligible for surgical intervention (2). In addition, consequent improvement in long-term survival post-surgery is only modest in HCC (3). Therefore, information related to the molecular changes associated with the development of HCC is critical to its successful prognosis.

Mounting evidence suggests that an inflammatory process is inherently associated with the development of HCC. The diseased liver gradually develops through a multi-step process. Several etiological factors, like hepatitis infection and heavy alcohol consumption, initially lead to cycles of liver injury or hepatocyte cell death and a compensatory regeneration (4). Under these circumstances, persistent inflammation is often elicited as an adaptive response to liver injury during the development of the disease. This is characterized by infiltration of immune cells followed by a perpetuation of healing response, eventually leading to subsequent liver fibrosis, cirrhosis, and ultimately HCC (5). Truly, HCC is a classic example of inflammation-associated cancer. Importantly, under these non-resolving inflammatory conditions, there is often de-regulated production of a vast array of cytokines, which augments the progression of HCC (6). For example, it triggers the generation of free radicals like reactive oxygen/nitrogen species (ROS/RNS) (7) and activation of key intrinsic factors like NFkB and STAT-3, which in concert with proliferation-induced mutagenesis not only fosters the tumor-promoting inflammatory microenvironment but also orchestrates stimulation of distinct neoplastic programs leading to subsequent progression of HCC (8). Given that HCC often manifests as an end-stage liver disorder, the complex set of interactions between the prevalent inflammatory mediators and the tumor cells in the HCC microenvironment is very intense and critical. Notably, during liver disease, chronic inflammation, profound tissue remodeling, and genetic alterations result in the production of both pro-inflammatory (e.g., IL-6, TNF-α) and/or anti-inflammatory or immuno-suppressive (e.g., TGF-β, IL-10) cytokines (9). However, the role of these cytokines is often overlapping, thus producing a complex web of interactions, which are poorly deciphered. Functional pleiotropy and redundancy of cytokines apparently pose a significant obstacle in elucidating the complex set of associations in the HCC microenvironment dictating the tumor cell fate. For example, multiple cytokines that bind to similar cell surface receptors can have analogous cellular effects reflecting apparent redundancy in a particular cellular context. Simultaneously, individual cytokines may often have multi-faceted functions in an organism in a cell type-specific manner depending on the presence or absence of other signaling components (10). Hence, understanding the key cytokine-related molecular signatures, and decoding the network and existing interactions between cytokines, is vital for developing an effective therapeutic approach against HCC.

One of the relevant features of HCC epidemiology is the disparity in the frequency of occurrence of the disease, which is three to five times more prevalent in males than females. This is often correlated with increased pro-inflammatory cytokine interleukin-6 (IL-6) in males (11). This cytokine is known to exert a pleiotropic effect in the liver. Classically, its role extends from infection defense and hepatocyte homeostasis to acting as a mitogen; however, an aberrant or persistent IL-6 signaling can lead to inflammatory conditions, metabolic abnormalities, or even malignancies of the liver (12). This places IL-6 at the crossroads of its conventionally believed hepato-protective role (13), compared to a cytokine involved in liver diseases (14). Therefore, extensive research has been channelized to decode the complex set of molecular events controlled by IL-6. The most accepted theory suggests that, in the inflamed liver microenvironment, the damaged hepatocytes release IL-1α that stimulates the Kupffer cells to secrete abundant IL-6, which thereafter binds to membrane-bound or soluble IL-6 receptors, along with signal transducer subunit gp130 to induce a classical or trans-signaling pathway, respectively (15). IL-6 subsequently can elicit its pro-proliferative effect by activating classical downstream targets like signal transducer and activator of transcription-3 (STAT-3). The spectrum of STAT activity may extend from stimulation of transcription of specific target genes, like Cyclin-D, Cyclin-B, Myc, or Bcl2, typically linked to cellular proliferation and survival (16–18), to induction in the expression of important epigenetic modulators like enhancer of zeste homolog 2 (EZH2) associated with the promotion of tumorigenesis and metastasis (19). No wonder an increased STAT-3 is detected in a vast majority of clinical HCC samples and is often positively correlated with tumor aggressiveness. Notably, apart from its canonical STAT-mediated signaling, IL-6 can also induce a non-canonical arm of signaling through activation of molecules like extracellular-signal-regulated kinases (ERKs) (20) or the Akt pathway, which helps in the transduction of IL-6-induced signals, especially its anti-apoptotic effects (21). Given the complex set of signals emanating post IL-6 binding to its receptor and its proven association with tumor enhancement, it demands further studies to understand its divergent downstream signaling and any cross-talk existing with other signaling factors predominant in HCC cells.

Herein, in a fibrotic or cirrhotic setting, when chronic liver damage overwhelms repair, there is often an increased production and bio-availability of the cytokine-transforming growth factor-beta (TGF-β), a crucial molecule enabled with the potential to modulate fibrosis (22). In the liver microenvironment, the cytokine is primarily secreted by the non-parenchymal cells like the hepatic stellate cells, Kupffer cells, and macrophages (23). The ligand is known to bind to its corresponding TGF-β receptor (TGF-βR), inducing phosphorylation and activation of downstream mediators like SMADs that induce transcription of specific target genes. Importantly, the biochemical function of TGF-β is often dynamic and alters as the tumor progresses. For example, during the early stages of cancer, it primarily acts as a tumor suppressor, typically by inducing cytostasis and apoptosis, while at later stages, TGF-β is known to promote multiple pro-tumorigenic features like epithelial to mesenchymal transition (EMT), invasion, metastasis, and angiogenesis. TGF-β plays this dichotomous role in hepatocarcinogenesis as well (24). Comparative functional genomic studies reveal that there might be two distinct groups of TGF-β-responsive genes, early and late, that can be triggered at different stages of liver cancer development, driving the conflicting effects (25). Furthermore, it has been observed that the expression level of diverse regulators of SMAD proteins like ELF and KLF17 that promote its cytostatic effect decreases as HCC progresses. In contrast, proteins like MUC1, TRPC6, and TRIM52, which facilitate the pro-tumorigenic function of SMADs, are over-expressed in advanced HCC (26). Therefore, TGF-β and its downstream signaling act as a critical switch, regulating HCC cell fate.

Given the relevance of the above cytokines in HCC development, it is critical to understand whether any cross-talk exists between components of IL-6 and TGF-β-induced signaling in HCC cells. Herein, our study provides critical insights into such possible molecular interactions, which can be modulated and essential for designing appropriate therapeutic strategies targeting HCC.

## Materials and Methods

### Chemicals and Reagents

Human TGF**-**β2 (#100-35B-10) was purchased from Peprotech; IL-6 (Sigma, #H7416), 3-[4,5-dimethylthiazol-2-yl]-2,5-di-phenyltetrazolium bromide (MTT) (SRL, #33611), Stattic (Sigma, #S7947), N-acetyl-L-cysteine (NAC) (SRL, #47866), H2-DCFDA (Sigma, #D6883), propidium iodide (PI) (Sigma, #P4864), and antifade mountant (4ʹ-6-diamidino-2-phenylindole, #P36962) were procured from Thermo Fisher Scientific. The primary antibodies used were PCNA (CST, #D2H8P XP), phospho-STAT-3 (CST, #D3A7 XP), total-STAT-3 (CST, #79D7), N-Cad (CST, #D4R1H XP), vimentin (CST, #D21H3 XP), SMAD2/3 (CST, #D7G7 XP), total SMAD2 (CST, #D43B4 XP), phospho-SMAD3 (CST, #C25A9), total SMAD3 (CST, #C67H9), phospho-NFκB-p65 (CST, #93H1), total NFκBp65 (CST, #D14E12 XP), EZH2 (CST, #D2C9), tri-methyl-histone H3 (Lys27) (CST, #C36B11), tri-methyl-histone H3 (Lys4) (CST, #C42D8), β-actin (BioBharati LifeScience, #BB-AB0024), and GAPDH (Santa Cruz, #sc-365062). Secondary antibodies included anti-rabbit (CST, #7074P2 and BB Lifescience, #BB SAB-01C) and anti-mouse (CST, #7076S). The enhanced chemiluminescence (ECL) kit was from Thermo Scientific (#32106); Alexa Fluor conjugated anti-rabbit IgG (#VI311611) was purchased from Invitrogen. The other reagents used were p65siRNA (CST, #6534S), SIS3 (BioVision, #SKU-2227), Lipofectamine (Invitrogen, #RNAiMax), IgG (Sigma, #I5006), magnetic beads (Bio-Rad, #Sure Beads Protein A Magnetic Beads 161-4013), and EZH2-inhibitor GSK-126 (Merck, #500580).

### Patient Data Analysis

To validate our data, we did an extensive literature survey to identify the altered levels of plasma/serum IL-6 or TGF-β in HCC patients. For this, we included different search strategies and used available literature databases such as PubMed, Scopus, Springer, Web of Science, OVID, Elsevier, Wiley, or ScienceDirect and looked for terms such as “IL-6”, “TGF-β”, “cytokines”, “liver cancer”, “HCC”, and/or “hepatocellular carcinoma” from the year 2000 to 2021. Our search for the data was based on the following criteria: (1) included studies on human subjects only and (2) studies reporting a mean or median cytokine level in HCC patients compared to healthy controls. These studies were executed in different parts of the world and on different sets of patients to check for the levels of cytokines in HCC patients (27–34). All these studies have followed a similar kind of workflow, which included collecting blood samples from patients and estimating the levels of cytokines by using ELISA. The baseline data are expressed as mean ± standard deviation (SD) and mean ± standard error (SE) deviation. Correlation between variables was assessed by Student’s t-test and Pearson correlation followed by two-tailed test. The three independent studies discussed in results, depicting high IL-6 levels, included the following: Study 1 consisting of 50 healthy controls and 238 HCC patients (35); Study 2 comprising of 24 healthy controls and 110 HCC patients (30); and Study 3 consisting of 30 healthy controls and 75 patients with HCC (36). In all studies, a p-value ≤ 0.05 is considered significant. For studies representing elevated levels of TGF-β, the given concentration of the cytokine was transformed to log values and expressed as mean ± standard deviation; a p-value ≤ 0.05 is considered significant. Herein, Study 1 had 9 healthy controls and 14 HCC patients (37); Study 2 consisted of 551 normal and 571 HCC samples (38); and Study 3 consisted of 30 healthy controls and 120 HCC patients (39). To ascertain the relevance of EZH2 in HCC, we analyzed the publicly available patient database and evaluated the transcript level of EZH2 genes. The Gene Expression Omnibus (GEO) database with accession ID GSE54238 was utilized for this analysis. It included expression profile for 10 normal livers (NL), 10 chronic inflammatory livers (IL), 10 cirrhotic livers (CL), 13 early HCC (eHCC), and 13 advanced HCC (aHCC) samples (40).

### Cell Culture and Treatments

Huh-7 (human hepatocellular carcinoma) was procured from the National Centre for Cell Science (NCCS), Pune. Cells were cultured at 5% CO_2,_ at 37°C in Dulbecco’s modified minimal essential medium (DMEM; Gibco, #12800-017) supplemented with 10% fetal bovine serum (FBS; Invitrogen, #26140-079) and antibiotics (1% penicillin–streptomycin solution; Invitrogen, #10378-016). Before cytokine exposure, Huh-7 cells were grown to ~70% confluency, media aspirated, rinsed with phosphate-buffered saline (PBS), and thereafter cultured in serum-starved medium (DMEM with 2% FBS) for 12 h (41). The cytokines used for treatment were IL-6 (25 ng/ml) or TGF-β (5 ng/ml), or a combination of both.

### Cell Viability Assay

Cell viability was assessed by MTT assay following procedures described earlier (42). Briefly, Huh-7 cells were cultured in 96-well plates and were serum-starved for 12 h; cells were then treated with different concentrations of cytokines. Following this, MTT (20 µl; stock concentration 5 mg/ml) was added and cells were incubated for 4 h. Viable cells were analyzed by dissolving formazan crystals with DMSO, post which readings were recorded at 570 nm with a differential filter of 630 nm by Multiskan Sky spectrophotometer (Thermo Scientific, #90273020). The percentage viability in treated samples is relative to that of control. The percentage of viable cells was calculated using the formula viability (%) = (mean absorbance value of drug-treated cells)/(mean absorbance value of control) * 100.

### RNA Isolation and cDNA Preparation

Total RNA extraction was done using TRIzol reagent (Sigma, #T9424). DNase I-treated (Thermo Scientific, #EN0521) RNA was used to synthesize first-strand complementary DNA (cDNA). cDNA was synthesized using an iScript cDNA synthesis kit (Bio-Rad, #1708891) with oligodT, as per the manufacturer’s protocol. Templates were amplified using gene-specific primers for PCNA (forward: 5′-TCACAGGGCAGTGTCTTCATT-3′; reverse: 5′-GGGTGACTGTAGCTGGGAAT-3′), Ki67 (forward: 5′-GACAGTACCGCAGATGACTC-3′; reverse: 5′-TACGTCCAGCATGTTCTGAGG-3′), vimentin (forward: 5′-TCTACGAGGAGGAGATGCGG-3′; reverse: 5′-GGTCAAGACGTGCCAGAGAC-3′), N-Cad (forward: 5′-CGAATGGATGAAAGACCCATCC-3′; reverse: 5′-GGAGCCACTGCCTTCATAGTCAA-3′), and IL-6R (forward: 5′-GGATGGTCAAGGACCTCCAG-3′; reverse: 5′-CTGGATTCTGTCCAAGGCGT-3′), taking β-actin (forward: 5′-CCACCATGTACCCTGGCATT-3′; reverse: 5′-CGGACTCGTCATACTCCTGC-3′) as housekeeping control, and detected using SYBR Green Supermix (Bio-Rad, #170-8882AP) in a CFX Connect RT-PCR system (Bio-Rad). The relative mRNA expression was calculated by Pfaffl’s method (43). The melting temperature for the PCRs was 56.4°C for PCNA, 56.7°C for Ki67, 60.4°C for vimentin, 61.4°C for N-Cad, 64.1°C for IL-6R, and 61.7°C for β-actin.

### Immunoblotting

Immunoblotting was performed following methods described elsewhere (44). Briefly, RIPA buffer (Sigma-Aldrich) was used to extract protein, and total protein was estimated using Bradford reagent (Sigma) at 595-nm wavelength. The cellular protein lysates were run in denaturing polyacrylamide gels and transferred to polyvinylidene fluoride membrane (PVDF, Bio-Rad), followed by blocking with 5% skimmed milk (HiMedia). Afterward, blots were probed and re-probed with specific primary antibodies (at dilution 1:1,000). The secondary antibodies (at dilution 1:2,000) were horseradish peroxide-conjugated and detected with a ECL detection system following the manufacturer’s protocol using ChemiDoc (Bio-Rad). Wherever required, the blots were cut to probe with different antibodies against proteins of different molecular weights. Differential protein expression was quantified using the ImageJ software and analyzed through the GraphPad Prism 8.0.1 software.

### Estimation of Intracellular Reactive Oxygen Species

Intracellular ROS levels were quantified using 2,7-dichlorofluorescein diacetate (H2DCF-DA) (Sigma). Cells were seeded at a density of 8 × 10^3^ cells/well in 96-well plates and exposed to the ROS scavenger NAC (10 mM) for 2 h prior to different cytokine treatments. In the case of STAT-3 inhibition-related ROS studies, Stattic was added after quenching ROS with NAC, followed by the addition of IL-6. Following exposure, the cells were washed with PBS and incubated with DCFDA (10 μM) for 1 h. Fluorescence was measured using a microplate reader at 485-nm excitation and 530-nm emission (Fluoroskan Ascent) (45).

### Analysis of Apoptotic Cells Using Propidium Iodide Uptake

Cells were seeded in 6-cm dishes at a 5 × 10^5^ cells/dish density. The following day, the cells were treated with the desired cytokine/pharmacological inhibitor for a specific time. After that, the cells were harvested, washed with PBS, and re-suspended in 500 μl of fresh PBS. PI was added and incubated for 20 min in the dark to detect the percentage of dead cells. After that, flow cytometric (CytoFLEX, Beckmann Coulter) analysis was performed, and the acquired data were analyzed using the CytExpert software (46).

### Cell Cycle Analysis

For cell cycle analysis, cells were seeded in 6-cm dishes. Post overnight incubation, cells were treated with cytokine for 72 h and harvested and washed with PBS followed by a spin at 5,000 rpm for 10 min at 4°C. After that, the pellet was re-suspended in a fixative (100 µl of PBS and 900 µl of ice-cold 70% ethanol), and the fixed cells were stored at 4°C overnight. Subsequently, cells were centrifuged and the pellet was re-suspended in 495 µl PBS with 5 µl of PI solution (2 mg/ml). The samples were then incubated in the dark for 10 min, followed by acquisition using a flow cytometer (CytoFLEX, Beckmann Coulter) and analysis using the CytExpert software (47).

### Immunofluorescence Microscopy

Cells were seeded on coverslips at 2.5 × 10^5^ cells/well and then treated with required concentrations of cytokines. Treated cells were then washed with 1X PBS and fixed with 4% paraformaldehyde, followed by incubation at room temperature for 10–15 min. After that, cells were treated with 0.2% Triton X for approximately 2 min. Post multiple PBS washes, cells were blocked with 2.5% bovine serum albumin (BSA) blocking solution for 1 h. The cells were then incubated overnight with primary antibody (1:500 dilution in 2.5% BSA) at 4°C, followed by washing twice with PBS, and then incubated with TRITC-conjugated secondary antibody (1:1,000 in 2.5% BSA) for 1 h. Coverslips were mounted on slides using antifade DAPI and imaged under a fluorescent microscope (Zeiss, Axio Scope A1, or Axio Observer.Z1/7). Images were analyzed using the Zen 3.2 (blue edition) software (48).

### Co-Immunoprecipitation Assay

Pre-washed magnetic beads were incubated with anti-STAT-3 antibody (1:50 dilution) for 1 h at 4°C. After three PBS-T washes, 500 µg of total protein extract was added and incubated overnight at 4°C. This was followed by removal of supernatant after magnetization and addition of 1X Laemmli buffer. Pre-heated protein samples were run on denaturing acrylamide gels, followed by transfer of protein on PVDF membrane and incubated with primary antibody overnight at 4°C. To normalize enrichment over STAT-3 antibody, IgG was used as a negative control.

### p65 Knockdown and STAT-3, SMAD3, and EZH2 Inhibition Studies

To knock down NFkB-p65, cells were stably transfected with SignalSilence NFκB p65siRNA II (CST, #6534S) using siRNA max (Sigma). Huh-7 cells were incubated with 100 nM p65siRNA (as per manufacturer’s instructions) for 6 h, followed by treatment of IL-6 for 72 h. For STAT-3 inhibition, cells were treated with 2 μM of STATTIC, 20 min prior to IL-6 treatment. For SMAD3 inhibition, Huh-7 cells were treated with 2.5 µM of SIS3, 2 h prior to TGF-β exposure; for EZH2 inhibition, cells were treated with GSK-126, 2 h prior to IL-6 exposure.

### Statistical Analysis

The data were analyzed using GraphPad Prism software version 8.0.1. The impact of specific treatment compared to control was statistically determined using Tukey’s test followed by one-way or two-way ANOVA. Multiple comparisons were analyzed using the Bonferroni method. Throughout, representative images are of experiments done in multiples and “Control” represents untreated cells. The symbols in parenthesis denote p-value significance; if the p-value is greater than 0.1, then the difference is considered not significant (ns), whereas if the p-value is ≤ 0.1, it is regarded as significant and denoted by the symbol * when ungrouped samples were compared or # when grouped samples were compared. The symbols **, ***, and **** represent a statistical significance of ≤0.01, ≤0.001, and <0.0001, respectively.

## Results

### De-Regulation of IL-6 and TGF-β in HCC Patient Datasets

**Figure 1:**
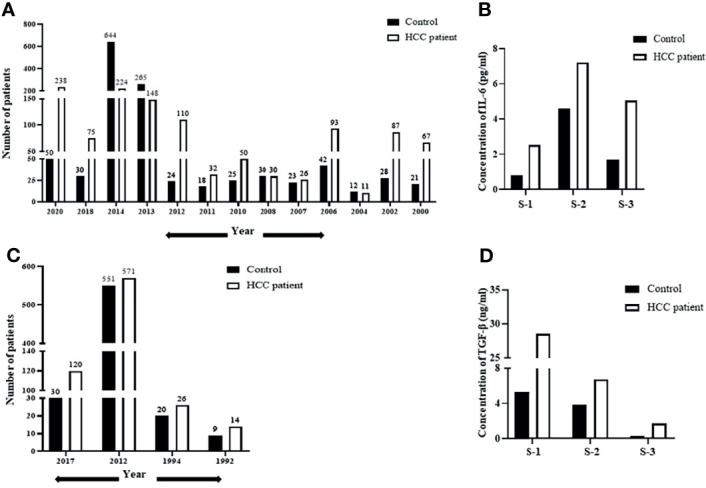
De-regulation of IL-6 and TGF-β in HCC patient datasets. **(A)** A year-wise timeline analysis showing IL-6 levels in serum/plasma of HCC patients compared to those in healthy control. **(B)** Analysis of concentration of IL-6 (pg/ml) in HCC patients from three independent studies compared to that in healthy control. **(C)** A year-wise timeline analysis showing TGF-β levels in serum/plasma of HCC patients compared to those in healthy control. **(D)** Analysis of concentration of TGF-β levels (ng/ml) in HCC patients compared to that in healthy control from three independent studies.

Human hepatocellular carcinoma is often associated with an altered expression of cytokines playing a critical role in the pathogenesis of the disease. To understand the relevance of IL-6 in liver diseases, we initially performed a screening of existing literature, published between 2000 and 2021, reporting altered blood levels of IL-6 in HCC patients compared to those in healthy control. Only the studies that report a mean or median cytokine level in HCC subjects compared to control were included for this analysis. Importantly, the year-wise analysis reveals that serum IL-6 levels are significantly higher in HCC patients (**Figure 1A**). A vast majority of these studies also propose IL-6 as a prognostic marker in HCC, and a high serum IL-6 level was correlated with tumor burden and aggressiveness (36). A bar graph representing the average levels of IL-6 concentration in HCC patients compared to those in healthy individuals from three independent studies is shown in **Figure 1B**. The level of IL-6 in the control group was 0.81 (0.06–5.75) pg/ml in comparison to 2.52 (1.40–4.99) pg/ml in HCC as per Study 1 (35), 4.61 (2.89–7.63) pg/ml in control to 7.21 (1.39–187.12) pg/ml in HCC as per Study 2 (30), and 1.69 (0.04–4.92) pg/ml in control to 5.06 (0.16–78.50) pg/ml in HCC in Study 3 (36). Our analysis, including studies conducted until 2020, clearly reinstates the importance of IL-6 in HCC and demands future detailed elucidation of its role in this context. Apart from IL-6, another cytokine that has been of clinical relevance in HCC is TGF-β (49). To appropriately understand the relevance of TGF-β as well in HCC, we performed a similar analysis like the above. We screened for literature reporting alteration of blood TGF-β levels in HCC patients. As evident from **Figure 1C**, the year-wise analysis of TGF-β levels in HCC patients compared to those in healthy control also shows a trend of increase in the diseased state (37–39). Three independent studies reporting plasma TGF-β levels in HCC patients, as represented in **Figure 1D**, also depict the prevalence of higher concentration of TGF-β in HCC patients. The level of TGF-β in the control group was 5.3 ± 3.3 (ng/ml) in comparison to 28.6 ± 3.3 (ng/ml) in HCC as per Study 1 (37), 3.58 ± 0.17log10 (pg/ml) in control to 3.83 ± 0.31 log10 (pg/ml) in HCC as per Study 2 (38), and 250.16 ± 284.61 (pg/ml) in control to 1,687.47 ± 1,462.81 (pg/ml) in HCC in Study 3 (39). Importantly, existing studies also show a significant co-existence of increased levels of both IL-6 and TGF-β in patients with liver diseases (50). Overall, the above analysis highlights the increased prevalence of these cytokines in patients suffering from HCC or liver diseases. Hence, analysis of any cross-talk existing between them becomes critical to understanding the disease and its subsequent management.

### IL-6 Induces Pro-proliferative Effects in Huh-7 Cells

**Figure 2:**
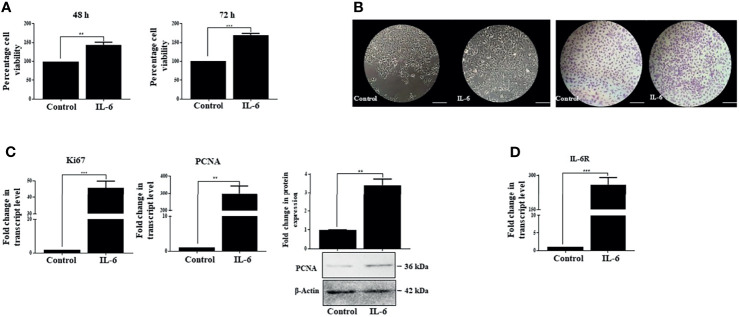
IL-6 induces pro-proliferative effects in Huh-7 cells. **(A)** Analysis of cell viability after 48 or 72 h post-IL-6 (25 ng/ml) exposure. **(B)** Phase-contrast and crystal violet stained images of untreated or IL-6-treated (25 ng/ml) cells at 72 h (scale bar: 100 μm). **(C)** Transcript levels of Ki67 and PCNA and protein level of PCNA after IL-6 treatment (25 ng/ml) for 72 h **(D)** The transcript level of IL-6R after IL-6 treatment for 72 h. ** and *** refers to p value significance of ≤0.01 and ≤0.001 respectively.

IL-6 is known to disseminate intracellular signals through its interaction with the IL-6R/gp130 receptor complex, which in turn initiates a series of downstream cascades canonically mediated by activated STAT-3. The latter then translocates to the nucleus and triggers transcription of a broad panel of target genes (51). Based on existing literature, it is understood that IL-6-mediated STAT-3 activation can elicit a pleiotropic effect, which may range from stimulation of proliferation, for example, in hepatocytes (3, 52), to its contrasting growth-suppressive effects in certain cell types like brain tumor, early-stage melanoma, breast carcinoma, or leukemia (53). Importantly, Moran et al. in 2008 also showed that IL-6 could induce a growth arrest in rat hepatoma cells in a STAT-3-dependent manner through regulation of CDK activity (17). Therefore, the contrasting downstream effects of IL-6 prompted us to analyze its role in human HCC cells Huh-7. Importantly, IL-6 exposure resulted in comparatively increased viability of cells with respect to untreated control, as measured through MTT assay at 48 and 72 h (**Figure 2A**). Furthermore, the phase-contrast images and crystal violet staining showed an increased cell number upon IL-6 treatment compared to untreated control after 72 h (**Figure 2B**). Simultaneously, enhanced transcript levels of known proliferation markers like Ki67 and PCNA were also observed in Huh-7 cells validating the growth-promoting effect of IL-6 (**Figure 2C**). In corroboration to the above, protein levels of PCNA also showed an increased expression upon the cytokine treatment (**Figure 2C**). Additionally, it was observed that IL-6 exposure could potentially result in an increased expression of the IL-6 receptor (IL-6R), thus putatively generating a feedback loop resulting in an amplification of IL-6-induced effect (**Figure 2D**). Existing studies indicate that IL-6 can stimulate EMT in cancer cells like cervical carcinoma or breast cancer (54). We observed a marginal change in EMT-associated markers like N-Cad and vimentin after 72 h of IL-6 treatment (**Supplementary Figure S1**), but a robust alteration was not observed. Overall, the above findings indicate the pro-proliferative functions of IL-6 in Huh-7 cells.

### Stattic, a STAT-3 Inhibitor, Generates ROS and Abrogates IL-6-Induced Proliferative Effects in Huh-7 Cells

**Figure 3:**
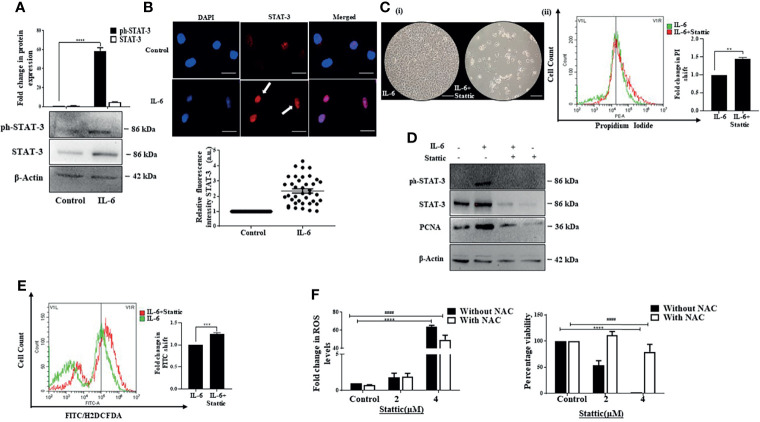
Stattic generates ROS and abrogates IL-6-induced proliferative effects. **(A)** Immunoblot showing expression of STAT-3 after 72 h of IL-6 exposure. **(B)** Immunofluorescence image and relative fluorescence intensity of STAT-3 in untreated and IL-6-treated cells (scale bar: 10 μm). The white arrow denotes STAT-3 protein. **(C)** (i) Phase-contrast image showing cells after IL-6, or IL-6 plus Stattic treatment post 24 h (scale bar: 100 μm); (ii) graph denoting PI fluorescence after IL-6, or IL-6 plus Stattic (2 μM) treatment post 24 h **(D)** Immunoblot showing expression of STAT-3 and PCNA in IL-6 and IL-6 plus Stattic (2 μM) treated cells after 24 h of exposure. **(E)** Graph representing a shift in green fluorescence indicating ROS levels post-Stattic (2 μM) treatment in IL-6 exposed cells after 24 h. **(F)** Analysis of intracellular ROS levels and corresponding cell viability after 24 h of Stattic exposure. **, *** and **** refers to p value significance of ≤0.01, ≤0.001 & ≤0.0001 respectively. #### represents a comparison between two groups with a significance of ≤0.0001.

Activated STAT-3 is the primary downstream effector that sustains IL-6-induced effects. We also observed an increase in phosphorylated STAT-3 upon exposure of Huh-7 cells to IL-6 (**Figure 3A**). In addition, a distinct increase in fluorescence intensity and nuclear localization of STAT-3 was observed after the cytokine treatment (**Figure 3B**). To understand whether STAT-3 is involved in stimulating cell proliferation, we inhibited STAT-3 with Stattic, a pharmacological inhibitor of STAT-3 that targets the STAT-3 SH2 domain, thereby blocking receptor dimerization (55). It resulted in a decreased cell viability, as evident from the phase-contrast images; an increase in PI-positive cells indicative of cell death (**Figure 3C**); and a decrease in IL-6-stimulated expression of PCNA, the marker associated with cellular proliferation, alongside its canonical effect on STAT-3 expression levels (**Figure 3D**). Furthermore, the mechanistic analysis revealed that Stattic exerts its effects through enhanced ROS levels in Huh-7 cells (**Figure 3E**). Inhibition of free radicals with the ROS quencher NAC led to reduced ROS and associated reduced sensitivity to Stattic, suggesting that ROS plays a pivotal role in Stattic-induced effects (**Figure 3F**). Overall, the above results confirm that STAT-3 mediates proliferative effects triggered by IL-6 in Huh-7 cells.

### p65 Activation Enhances Proliferation Co-Operatively With STAT-3

**Figure 4:**
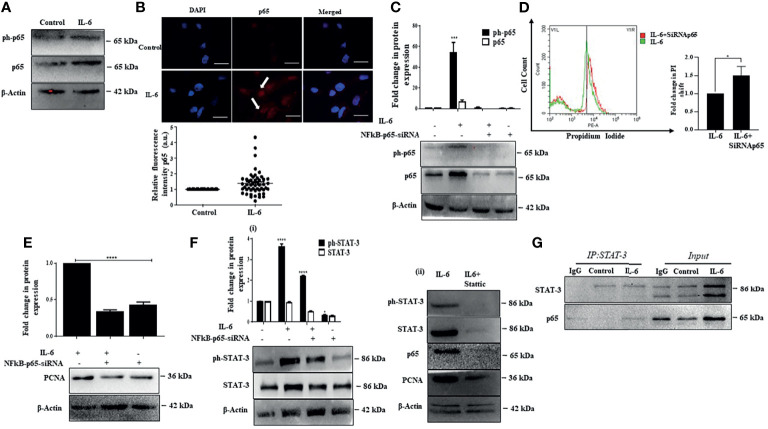
p65 activation enhances proliferation co-operatively with STAT-3. **(A)** Immunoblot showing expression of p65 after IL-6 treatment for 72 h. **(B)** Immunofluorescence image and relative fluorescence intensity of p65 in untreated and IL-6-treated cells (scale bar: 10 μm). The white arrow indicates p65. **(C)** Immunoblot showing expression of p65 in IL-6-treated or IL-6 plus p65-siRNA treated cells after 72 h of exposure. **(D)** Graph representing a shift in PI fluorescence in IL-6-treated or IL-6 plus p65-siRNA treated cells after 72 h. **(E)** Immunoblot showing expression of PCNA in IL-6-treated or IL-6 plus p65-siRNA treated cells after 72 h of treatment. **(F)** (i) Immunoblot showing expression of STAT-3 in IL-6-treated or IL-6 plus p65-siRNA treated cells after 72 h (ii) Immunoblot showing expression of STAT-3, p65, and PCNA in IL-6-treated or IL-6 plus Stattic exposed cells. **(G)** Co-immunoprecipitation analysis of STAT-3 with p65 after IL-6 exposure for 72 h The proteins were immunoprecipitated with anti-STAT-3 antibody and then probed with p65. *, *** and **** refers to p value significance of ≤0.1, ≤0.001 & ≤ 0.0001 respectively.

Existing studies suggest that several signaling pathways are involved in liver injury, inflammation, and regeneration response and play a vital role in HCC development. Notably, one of the critical pathways activated during liver injury and inflammation and plays a predominant role in liver carcinogenesis is the classical NFκB signaling (56). Moreover, both IL-6-induced STAT-3 and NFκB are engaged in cross-talk. They can control the expression of a host of downstream targets that can regulate cell proliferation, survival, stress responses, and immune functions (57). Herein, the p50/p65 heterodimer from the NFκB family is the most predominant transcription complex that mediates NFκB functions (58). In our study, we observed that IL-6 causes an increase in p65 protein levels along with an increase in intracellular p65 fluorescence intensity, suggesting induction of NFκB signaling (**Figures 4A, B**). We were interested to understand the role of p65 in Huh-7 cells. Interestingly, a siRNA-mediated ablation of p65 (**Figure 4C**) resulted in induction of cell death in IL-6-stimulated Huh-7 cells as analyzed through PI staining (**Figure 4D**). Furthermore, an inhibition of p65 also resulted in reduced expression of IL-6-induced proliferation markers, like PCNA indicating that p65 is primarily involved in the induction of cellular proliferation of Huh-7 cells (**Figure 4E**). Since STAT-3 primarily mediates IL-6-induced effects, we further analyzed the effects of p65 ablation on STAT-3 and vice versa. Interestingly, siRNA-mediated knockdown of p65 resulted in a reduction in STAT-3 levels, suggesting a feedback loop existing between p65 and STAT-3 (**Figure 4F**). On a similar note, Stattic-mediated inhibition of STAT-3 also caused a reduction in p65 protein levels accompanied by a reduction in levels of PCNA protein (**Figure 4F**). These results indicate an intense cross-talk between IL-6-induced STAT-3 and p65 regulating the proliferation of Huh-7 cells. However, whether p65 and STAT-3 physically interact to control cellular proliferative functions was not clear. Interestingly, co-immunoprecipitation studies confirm that p65 and STAT-3 proteins physically interact, thus regulating the fate of Huh-7 cells (**Figure 4G**).

### IL-6 Exposure Has Minimal Effect on TGF-β-Induced SMAD-Dependent EMT

**Figure 5:**
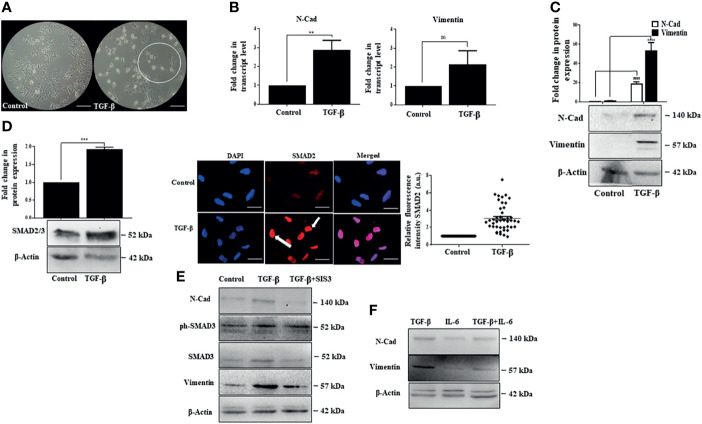
IL-6 fails to enhance TGF-β-induced SMAD-dependent EMT. **(A)** Phase-contrast images of TGF-β treated cells after 72 h of exposure compared to those of untreated control (scale bar: 100 μm). The encircled area represents morphologically distinct cells. **(B)** Transcript levels of N-Cad and vimentin after TGF-β treatment compared to those of untreated control. **(C)** Immunoblot showing expression of N-Cad and vimentin after TGF-β exposure for 72 h. **(D)** Immunoblot showing expression of SMAD2/3 after TGF-β treatment for 72 h; immunofluorescence image and relative fluorescence intensity of SMAD2 after TGF-β treatment (scale bar: 10 μm). The white arrow denotes SMAD2 protein. **(E)** Immunoblot showing SMAD3, N-Cad, and vimentin expression in cells treated with TGF-β or TGF-β plus SIS3 for 72 h **(F)** Immunoblot showing expression of N-Cad and vimentin after exposure to TGF-β or IL-6 or in combination for 72 h. **, *** and ns refers to p value significance of ≤0.01, ≤0.001 & not significant respectively.

While IL-6 was found to have a growth stimulatory effect, we were interested in analyzing the impact of TGF-β, a cytokine abundantly produced in the HCC microenvironment (24). It is well known that TGF-β induces epithelial to mesenchymal transition (EMT), facilitating tumor cell invasion (59). In corroboration to the above, distinct elongated morphology of cells was observed post-TGF-β exposure in Huh-7 cells (**Figure 5A**). This was associated with an increase in transcript and protein levels of EMT-associated markers like N-Cad and vimentin (**Figures 5B, C**). Classically, TGF-β signaling is known to activate intracellular molecules, like SMAD2 and SMAD3, to drive EMT features and associated transcriptional programs. In accordance with the above, we observed a substantial increase in protein levels of SMAD2/3 along with an increased nuclear internalization of the SMAD2 upon the addition of TGF-β in Huh-7 cells (**Figure 5D**). In addition to the SMADs, the cytokine TGF-β can utilize many signaling pathways to regulate a wide array of cellular functions, including EMT. To ascertain that TGF-β-induced EMT in Huh-7 cells is mediated by the classical SMAD-dependent pathway in Huh-7 cells, we inhibited SMAD signaling with SIS3, a pharmacological inhibitor. This resulted in a substantial decrease in N-Cad and vimentin protein expression, suggesting that SMAD proteins regulate the expression of EMT markers in Huh-7 cells (**Figure 5E**). Given that tumor cells constantly interact with multiple cytokines simultaneously present in the TME, inducing a complex web of cross-talk between different pathways induced by each cytokine, we were interested to understand the effect of simultaneous administration of IL-6 and TGF-β in Huh-7 cells. While earlier reports suggest that IL-6 can independently induce EMT-like features in multiple cancer types (60), in our study, at the stipulated dose, simultaneous addition of IL-6 with TGF-β did not enhance a robust EMT feature, as evident from the expression of EMT-associated markers (**Figure 5F**). This suggests that IL-6, though it has a positive influence on cellular proliferation, has minimal effect on EMT when administered with TGF-β in Huh-7 cells. Herein, we were interested to understand the impact of simultaneous administration of the cytokines on cellular proliferation.

### IL-6-Induced Cell Proliferation Is Attenuated by TGF-β

**Figure 6:**
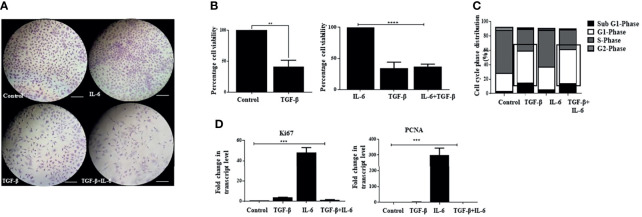
IL-6-induced cellular proliferation is attenuated by TGF-β. **(A)** Crystal violet staining after exposure to TGF-β or IL-6 or TGF-β plus IL-6 for 72 h compared to untreated control (scale bar: 100 μm). **(B)** Analysis of cell viability after TGF-β/IL-6 or TGF-β plus IL-6 treatment for 72 h. **(C)** Analysis of cell cycle stages after treatment with TGF-β/IL-6 or TGF-β plus IL-6 for 72 h. **(D)** Transcript levels of Ki67 and PCNA after TGF-β/IL-6 or TGF-β plus IL-6 treatment for 72 h. **, *** and **** refers to p value significance of ≤0.01, ≤0.001 & ≤0.0001 respectively.

Interestingly, exposure of Huh-7 cells to TGF-β alone showed reduced proliferation of cells (**Figures 6A, B**). In this regard, TGF-β has been previously attributed to cellular growth arrest-like functions in mammalian cells (61); however, when both IL-6 and TGF-β were administered together, we observed that the TGF-β-induced effects predominate. Despite the presence of IL-6 in media, an attenuated proliferative potential was observed (**Figure 6A**). Analysis of cell viability through MTT assay revealed a decreased percentage of cells upon TGF-β treatment, which persisted upon the combination treatment, suggesting the prevalence of TGF-β-associated features when both the cytokines are administered together (**Figure 6B**). Besides, the cells treated with IL-6 retained EMT-like morphological features in the presence of TGF-β (not shown). Furthermore, to understand whether the cells enter a probable arrest of cell division, we performed flow cytometry-based analysis, which showed an increase and a comparable percentage of cells at the G_1_ phase of the cell cycle upon TGF-β or TGF-β plus IL-6 treatment (**Figure 6C**). Additionally, there was a decreased transcript level of IL-6-induced proliferation markers like Ki67 or PCNA when TGF-β was added in culture (**Figure 6D**). The above observations indicate that the cellular effects of TGF-β predominate in the presence of IL-6, resulting in attenuation of IL-6-induced proliferation of tumor cells.

### IL-6-Induced Effects Are Suppressed by TGF-β Through Inhibition of p65 and STAT-3

**Figure 7:**
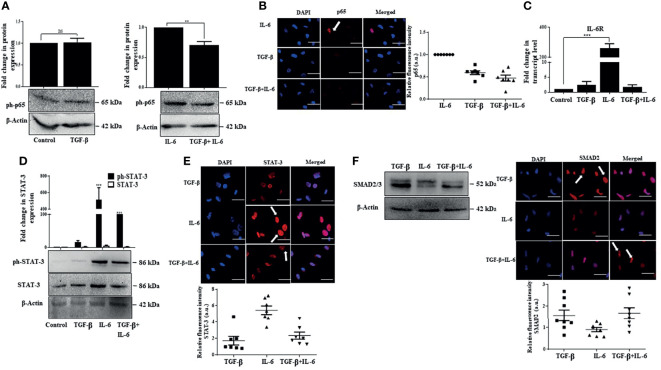
IL-6-induced effects are suppressed by TGF-β through inhibition of p65 and STAT-3. **(A)** Immunoblot showing expression of phosphorylated p65 after TGF-β/IL-6 or TGF-β plus IL-6 treatment for 72 h. **(B)** Immunofluorescence image and relative fluorescence intensity of p65 in TGF-β/IL-6 or TGF-β plus IL-6 treated cells (scale bar: 10 μm). White arrow indicates p65 protein. **(C)** Transcript levels of IL-6R after TGF-β/IL-6 or TGF-β plus IL-6 treatment for 72 h. **(D)** Immunoblot showing expression of STAT-3 in TGF-β/IL-6 or TGF-β plus IL-6 treated cells after 72 h of exposure. **(E)** Immunofluorescence image and relative fluorescence intensity of STAT-3 in TGF-β/IL-6 or TGF-β plus IL-6 treated cells (scale bar: 10 μm) White arrow indicates STAT-3 protein. **(F)** Immunoblot showing expression of SMAD2/3 after TGF-β/IL-6 or TGF-β plus IL-6 treatment for 72 h; immunofluorescence image and relative fluorescence intensity of SMAD2 in TGF-β/IL-6 or TGF-β plus IL-6 treated cells (scale bar: 10 μm). The white arrow indicates SMAD2 protein. **, *** and ns refers to p value significance of ≤0.01, ≤0.001 & not significant respectively.

We were thereafter interested to understand the mechanism by which TGF-β suppresses IL-6-induced effects. One of the salient features observed in our study was the activation of p65 by IL-6, which positively regulates cellular proliferation. Notably, a dampened IL-6-induced p65 protein expression along with a reduction in p65 fluorescence intensity was observed upon concomitant addition of TGF-β in Huh-7 cells (**Figures 7A, B**). This implicates that TGF-β can suppress p65 signaling resulting in reduced proliferation of cells. Furthermore, while there was an increase in expression of IL-6 receptor (IL-6R) upon addition of only IL-6 on Huh-7 cells, in contrast, simultaneous addition of both the cytokines resulted in a reduced expression of IL-6R, thus attenuating IL-6-induced signaling (**Figure 7C**). Consequently, we further observed that TGF-β results in decreased IL-6-induced STAT-3 activation and its nuclear localization (**Figures 7D, E**); however, SMAD expression and its localization remained more or less unchanged upon IL-6 addition (**Figure 7F**). Overall, it can be concluded that TGF-β can inhibit IL-6-induced signaling and its subsequent effects by regulating IL-6R and subsequent downstream signals.

### IL-6 Induces Open Chromatin Mark While TGF-β Promotes Closed Chromatin Signature

**Figure 8:**
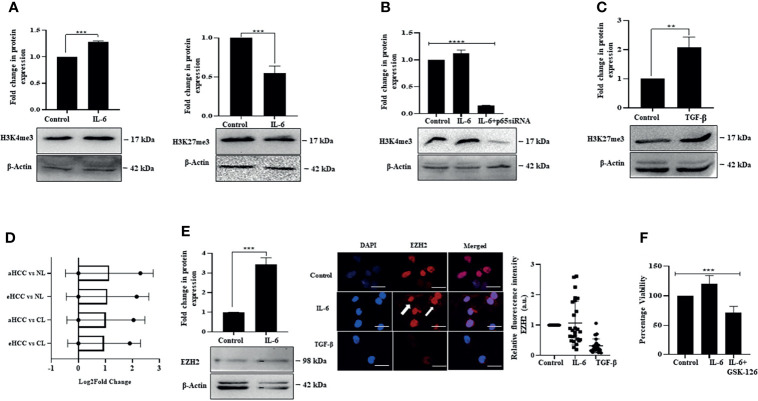
IL-6 induces open chromatin marks while TGF-β promotes closed chromatin signature. **(A)** Immunoblot showing expression of H3K4me3 and H3K27me3 in IL-6-treated cells post 72 h of exposure. **(B)** Immunoblot showing expression of H3K4me3 in untreated or IL-6-treated or IL-6 plus p65-siRNA treated cells at 72 h. **(C)** Immunoblot showing expression of H3K27me3 after TGF-β treatment for 72 h. **(D)** EZH2 transcripts levels obtained from the GEO database for subjects with cirrhotic liver (CL), early HCC (eHCC), and advanced HCC (aHCC) compared to healthy control (normal-NL). **(E)** Immunoblot showing expression of EZH2 in IL-6-treated cells at 72 h; immunofluorescence image and relative fluorescence intensity of EZH2 after IL-6 or TGF-β treatment (scale bar: 10 μm). The white arrow indicates EZH2 protein. **(F)** Cell viability analysis after IL-6 or IL-6 plus GSK-126 (50 µM) treatment for 72 h. **, *** and **** refers to p value significance of ≤0.01, ≤0.001 & ≤0.0001 respectively.

We were thereafter interested to understand whether IL-6-induced proliferation and TGF-β-induced arrest involve regulation of the epigenome or not. The epigenetic events primarily manifest through histone modifications, which allow or inhibit the accessibility of genomic regions to transcriptional events, depending on the type of modifications. Interestingly, we observed that IL-6 induced pro-proliferative effects were associated with enrichment of global histone-3 lysine-4 trimethylation (H3K4me3) mark, considered as an open chromatin tag conducive to transcription of genes; however, there was a minimal alteration of histone-3 lysine-27 trimethylation (H3K27me3) mark, a closed chromatin signature (**Figure 8A**). Importantly, a siRNA-mediated ablation of IL-6-induced p65 resulted in a reduced H3K4me3 mark, suggesting an intense cross-talk between the IL-6-induced intracellular signaling and regulation of the epigenetic signatures (**Figure 8B**). In contrast to IL-6-induced effects, TGF-β exposure to Huh-7 cells resulted in an increase in closed chromatin mark H3K27me3, suggesting that TGF-β-induced growth arrest is coupled to repression of transcription through enrichment of overall repressive chromatin tags (**Figure 8C**). Herein, hyperactivation of STAT-3 has been associated with enhanced EZH2 expression resulting in a poor prognosis of multiple cancers types like gastric carcinoma (19). In corroboration to the above, analysis of an available HCC patient dataset from the GEO portal showed an upregulation in transcript levels of EZH2 in patients with early or advanced stage HCC compared to normal (**Figure 8D**). On a similar note, in our *in vitro* study, IL-6 exposure to Huh-7 cells resulted in an increased expression of EZH2 alongside enhancement of intracellular fluorescence intensity of the protein (**Figure 8E**). Since EZH2 upregulation has been earlier correlated with tumor progression (62), we apprehend that an increase in EZH2 in conjunction with enrichment of open chromatin mark, as observed in Huh-7 cells, is associated with its pro-proliferative effects. Importantly, pharmacological inhibition of EZH2 activity by GSK-126 resulted in a reduction in IL-6-induced cell proliferation (**Figure 8F**). Further studies are required in this direction to understand the specific genes regulated by the chromatin marks.

## Discussion

A complex network of interactions exists between cytokines in the tumor microenvironment, contributing to the evolution of a favorable niche for tumor development (6). Here, each cytokine can trigger a prominent signaling cascade, which can be intruded by signaling induced by the other cytokines (63). Understanding the fundamental functional interactions between the cytokines might hence be critical to mapping the course of the disease and in identifying putative targets for future therapy. Therefore, cross-talk between the cytokines in various settings is an intense area of research. In this context, liver cancer is known to be invariably associated with an inflammatory setting (64) and deserves special attention. Under the inflammatory conditions, factors that were found to be abundantly expressed in patients with HCC included IL-6 and TGF-β. Compelling evidence suggests that they are also associated with the poor prognosis of patients with HCC (65). Therefore, this study was designed to gain enhanced understanding of the cross-talk between signaling cascades associated with exposure to these cytokines, which is of significant value for the development of approaches for inhibiting HCC progression.

While there are ample reports on IL-6 and TGF-β independently contributing to the progression of multiple cancer types, earlier studies by Yao et al. (2010) on lung cancer cells show that factors like IL-6 and TGF-β can act in unison prominently contributing toward resistance to chemotherapy. Herein, TGF-β was also stimulating IL-6 secretion, resulting in an adaptive mechanism that prevents targeted therapy (66). On a similar note, Yamada et al. also reported a high expression of IL-6 and TGF-β in biliary tract cancer cells. It showed an intense cross-talk between these inflammation-associated cytokines, which positively correlated with cellular invasion, EMT, and resistance (67). Though reports are more inclined toward synergism between these two cytokines promoting tumor development, our observations contradict this paradigm. We observed that at the stipulated dose and conditions, TGF-β could attenuate IL-6-induced pro-proliferative effects. Importantly, in corroboration to our findings, Yamamoto et al. in 2001 observed that, though TGF-β can significantly stimulate IL-6 signaling and STAT-3-dependent reporter activity, however, intriguingly, this effect is suppressed after prolonged exposure to the cytokine (68). Additionally, earlier studies also show that TGF-β can suppress the basal expression of several genes in hepatoma cells and can even potentially inhibit few IL-6-induced genes, which is important from the perspective of manipulating cytokine balance as a prospective therapeutic strategy (69). We apprehend that these cytokines have a pleiotropic effect in the cellular system and therefore can arguably impart opposite effects for dose, context, time, and cellular systems.

**Figure 9:**
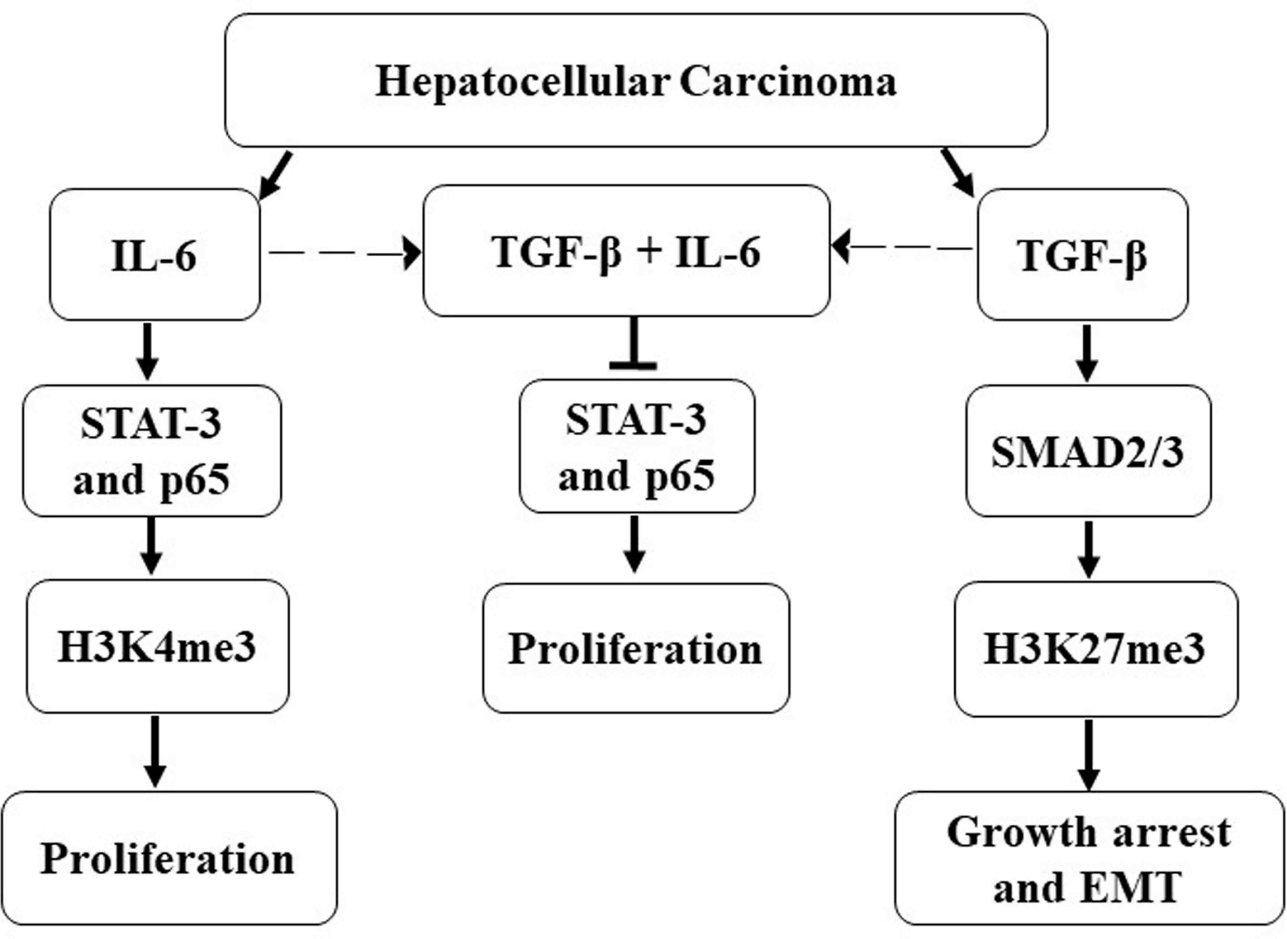
Schematic representation of crosstalk between IL-6 and TGF-β signaling.

Induction of EMT is a well-known manifestation of primarily TGF-β response. However, IL-6 has also been reported with similar activities (54). Similarly, IL-6 has been predominantly associated with proliferation signaling (70). Herein, we show that cellular programs that correspond to mesenchymalization and proliferation are distinct, and concurrent exposure of HCC cells does not necessarily result in enhanced proliferation and/or a massive alteration of EMT-associated features. Interestingly, we observed a decrease in expression of IL-6R transcript levels post TGF-β treatment which corroborates with studies by Wiegertjes et al. that portrays a similar phenomenon in chondrocytes (71). Herein, several studies mention the co-operation of NFκB pathway as a non-canonical arm of TGF-β signaling inducing or maintaining invasive features in multiple cancers (72); paradoxically, TNF-α-induced NFκB has been found to inhibit SMAD-dependent TGF-β activity as well (73). Interestingly, we found that the p65 subunit of the NFκB pathway is induced by IL-6 and is required for the proliferation of Huh-7 cells, and a simultaneous TGF-β exposure abrogates this effect. Additionally, we show that the effect of these cytokines is not restricted to the receptor regulation or downstream signaling events but culminates/originates at the epigenomic level as well. In this regard, earlier studies have demonstrated that epigenetic regulation is an integral part of TGF-β signaling. However, it remains a critical dimension that is yet to be fully elucidated. Herein, the SMADs are key transcription factors that can recruit various epigenetic regulators to orient the transcriptome (74). For example, TGF-β-induced SMAD3/4 mediates acetylation or methylation at H3K9 during EMT in breast cancer cells, thus activating genes like SNAIL or repressing genes like E-cadherin, respectively (74). In our study, we observed that IL-6 induces enrichment of open chromatin marks (H3K4me3) along with EZH2 expression, while, in contrast, TGF-β predominantly results in closed chromatin signatures (H3K27me3) coupled to growth arrest. Our preliminary data show that both IL-6 and TGF-β serve as versatile molecules in fine-tuning mechanisms for transcriptional regulation through epigenetics. However, the genes regulated by the epigenetic marks remain further elucidated. The overall observation from the study is represented in a schematic diagram (**Figure 9**).

Since HCC has a close association with IL-6 and its poor prognosis, many modalities have been carried out to mitigate the effects of IL-6-induced tumor progression. In this regard, the contribution of potent cytokines in such interactions is gradually getting uncovered. Herein, signaling-based antagonist approaches are relatively less explored or poorly understood. Hence, our study provides novel insights into alternative methods and portrays probable mechanisms that may be tinkered or targeted to prevent HCC development.

## Data Availability Statement

The original contributions presented in the study are included in the article/**Supplementary Material**. Further inquiries can be directed to the corresponding author.

## Author Contributions

AS: data curation, formal analysis and methodology, original draft preparation. HS: data curation, software, methodology. SK and TSB: Formal analysis and reviewing. SC: in-silico data curation, editing software and reviewing. RC: Formal analysis, data curation, writing and reviewing. SM: Funding acquisition, data curation, writing, editing, reviewing, project administration and resources. All authors contributed to the article and approved the submitted version.

## Funding

This research was funded by DST-SERB grant number EMR/2017/004149 and partially funded by CSIR GRANT 37(1723)/19/EMR II DATED 15.5.2019.

## Conflict of Interest

The authors declare that the research was conducted in the absence of any commercial or financial relationships that could be construed as a potential conflict of interest.

## Publisher’s Note

All claims expressed in this article are solely those of the authors and do not necessarily represent those of their affiliated organizations, or those of the publisher, the editors and the reviewers. Any product that may be evaluated in this article, or claim that may be made by its manufacturer, is not guaranteed or endorsed by the publisher.

## Supplementary Material

Supplementary Figure S1 Effect of IL-6 on EMT markers in Huh-7 cells.
