# Supplementary figures and images for: Interleukin-6 Induced Proliferation Is Attenuated by Transforming Growth Factor-β-Induced Signaling in Human Hepatocellular Carcinoma Cells
Source: Front Oncol. 2022 Jan 20;11:811941. doi: 10.3389/fonc.2021.811941 (PMC8810489; doi:10.3389/fonc.2021.811941)

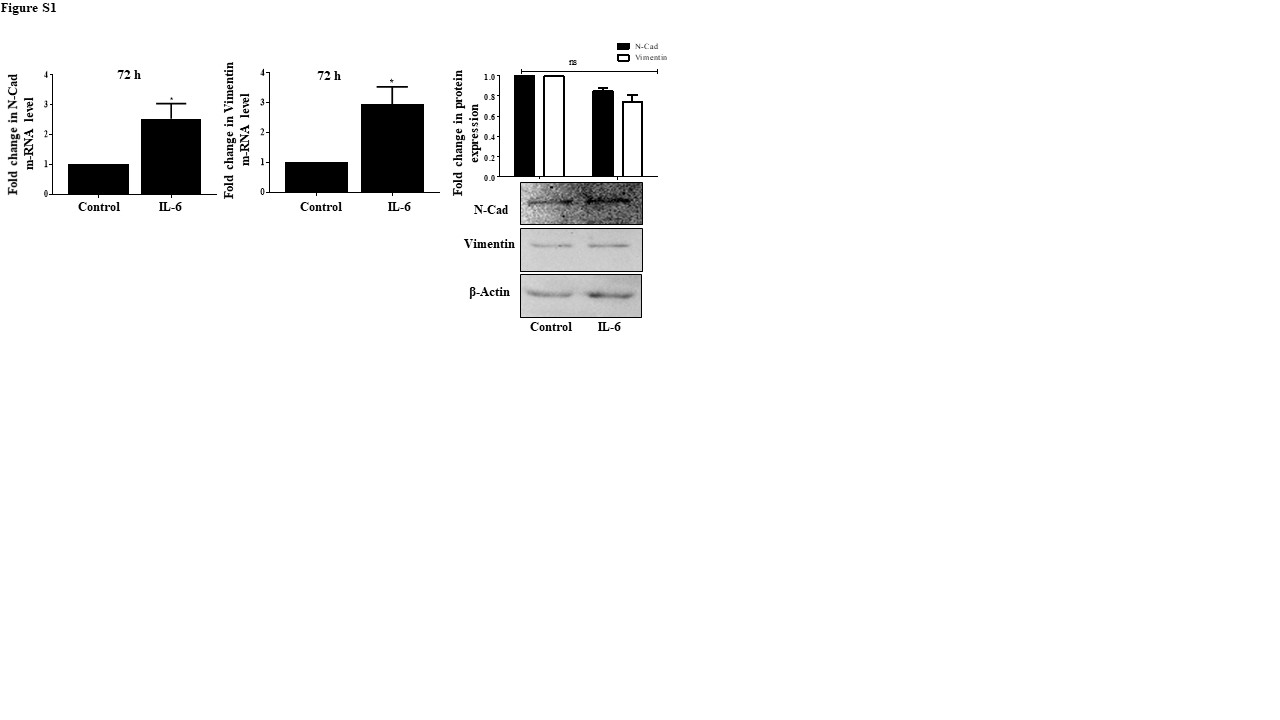

Supplement: Supplementary Figure S1 — Effect of IL-6 on EMT markers in Huh-7 cells. [file Image_1.jpeg]
