# Supplementary material for: Interleukin-6 Induced Proliferation Is Attenuated by Transforming Growth Factor-β-Induced Signaling in Human Hepatocellular Carcinoma Cells
Source: Front Oncol. 2022 Jan 20;11:811941. doi: 10.3389/fonc.2021.811941 (PMC8810489; doi:10.3389/fonc.2021.811941)
Supplement: Supplementary file 2 [file DataSheet_1.pdf]

Figure: 2

a. **Cell**: Huh7 **Cytokine**: IL-6 **Time point**: 48 hours

Experiment: MTT Assay

	A	B
	Control	IL-6
	Y	Y
1	100.000000	131.492000
2	100.000000	156.086200
3	100.000000	143.651000

a . **Cell**: Huh7 **Cytokine**: IL-6 **Time point**: 72 hours

Experiment: MTT Assay

	A	B
	Control	IL-6
	Y	Y
1	100.0000	161.748100
2	100.0000	169.630000
3	100.0000	177.568900

c. **Cell**: Huh7 **Cytokine**: IL-6 **Time point**: 72 hours

Experiment: RT-PCR:Ki67

	A	B
	Control	IL-6
	Y	Y
1	1.00	224.710
2	1.00	376.100
3	1.00	300.405

c. **Cell**: Huh7 **Cytokine**: IL-6 **Time point**: 72 hours
Experiment: RT-PCR:PCNA

	A	B
	Control	IL-6
	Y	Y
1	1.00	38.97
2	1.00	52.95
3	1.00	45.96

c. **Cell**: Huh7 **Cytokine**: IL-6 **Time point**: 72 hours

Experiment: Western Blotting : PCNA(CST), β-Actin(Biobharati)

Control IL-6

PCNA 30kDa

β-Actin 42 kDa

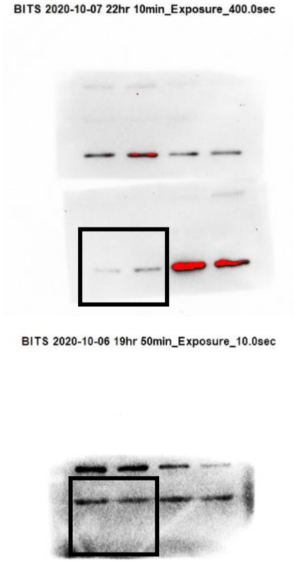

d. **Cell**: Huh7 **Cytokine**: IL-6 **Time point**: 72 hours

Experiment: RT-PCR:IL-6R

	A	B
	Control	IL-6
	Y	Y
1	1.00	207.72
2	1.00	313.12
3	1.00	
4	1.00	260.42

Figure3

a. Cell: Huh7 Cytokine: IL-6 Time point: 72 hours

Experiment: Western Blotting: ph-STAT3(CST), Total STAT3(CST) β-Actin(Biobharati)

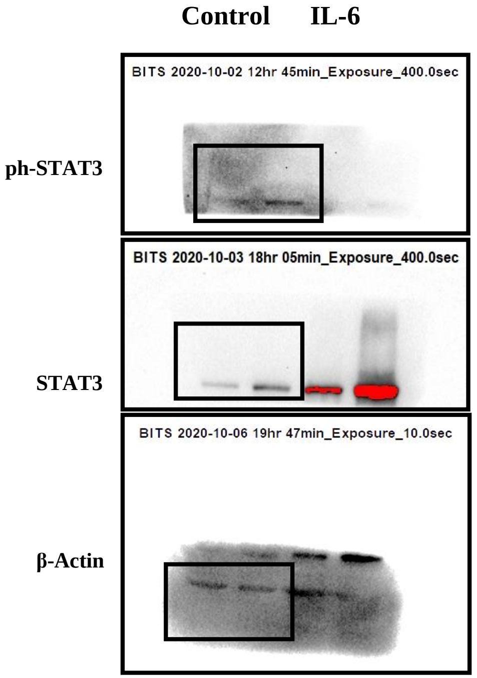

(b) Cell: Huh7 Cytokine: IL-6 Time point: 4 hours Experiment: Immunofluorescence Total STAT3(CST)

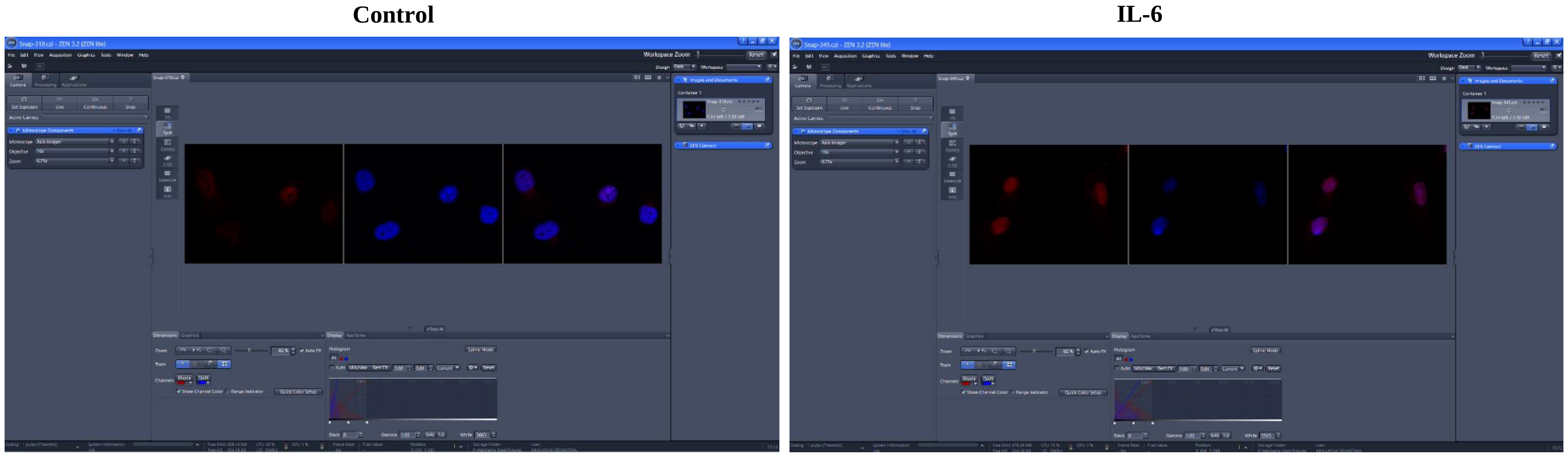

(c) (ii) Cell: Huh7 Cytokine: IL-6 and Stattic Time point: 24 hours Experiment: Annexin-PI (Flow cytometry)

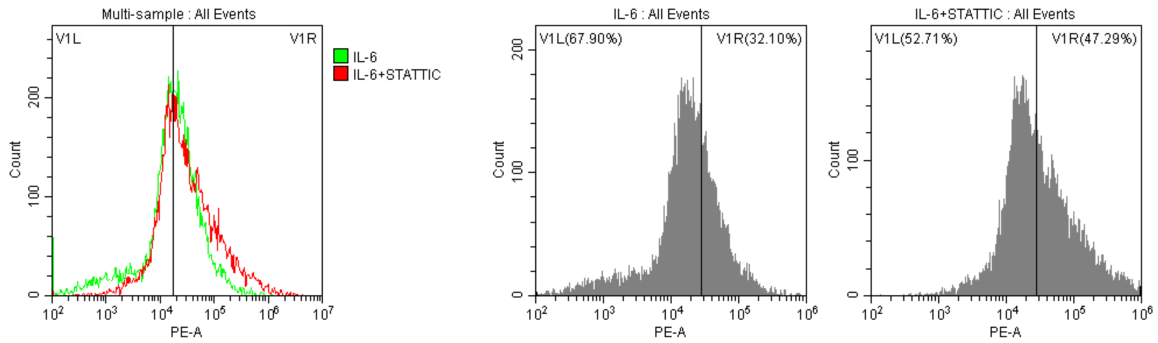

Continued on the next slide

d. Cell: Huh7 Cytokine: IL-6 Time point: 72 hours

Experiment: Western Blotting: ph-STAT3(CST), Total STAT3(CST),PCNA(CST) β-Actin(Biobharati)

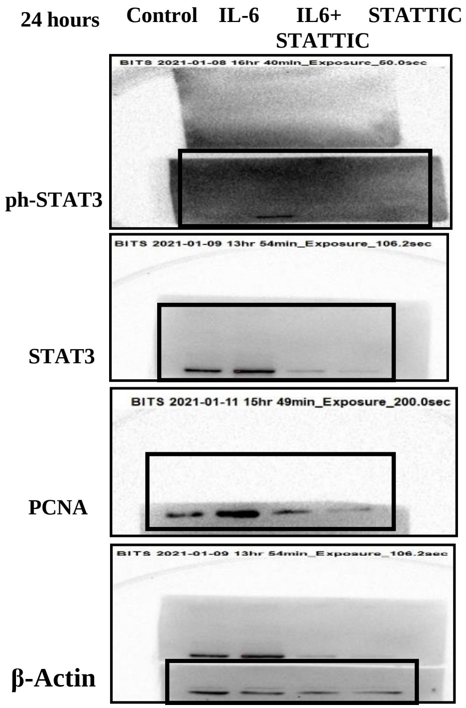

e. Cell: Huh7 Cytokine: IL-6 and Stattic Time point: 24 hours

Experiment: Flow cytometer: DCFDA:ROS

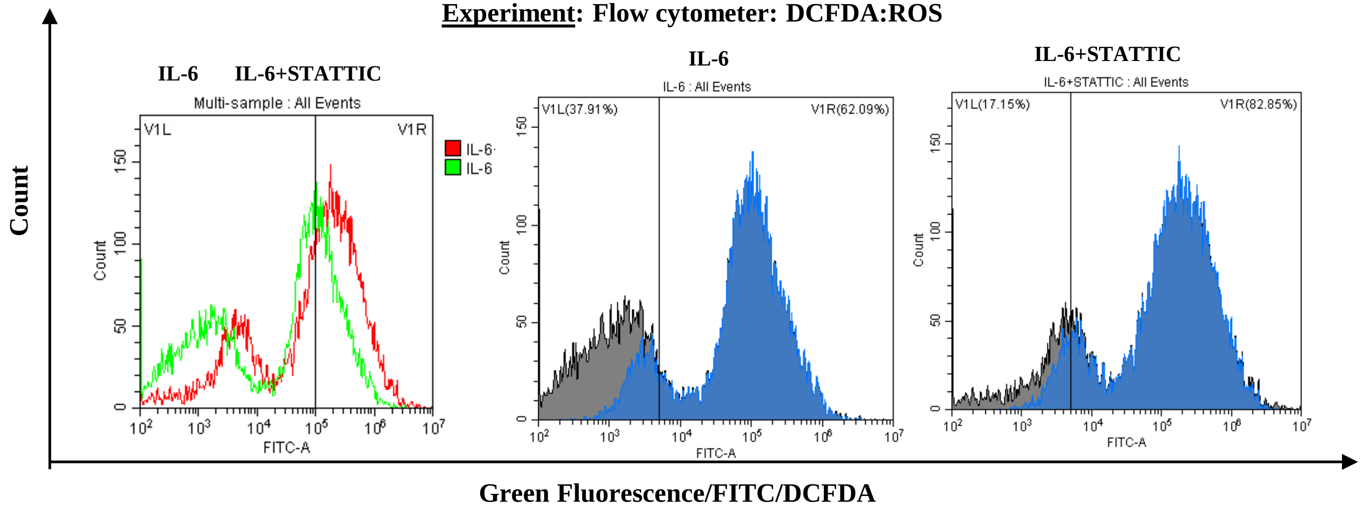

f. Cell: Huh7 Cytokine: IL-6 and STATTIC Time point: 24 hours

Experiment: DCFDA:ROS

Table format: Grouped	A				B			
	Without NAC				With NAC			
	A:Y1	A:Y2	A:Y3	A:Y4	B:Y1	B:Y2	B:Y3	B:Y4
1 Control	1.000000	1.000000	1.000000	1.000000	0.768036	0.657340	0.703623	0.848945
2 2uM Stattic	3.497688	1.426858	1.476078	0.787173	2.170717	2.955885	1.701640	0.802135
3 4uM Stattic	62.401850	63.371990	66.608290		42.086380	59.034630	47.046880	

F. Cell: Huh7 Cytokine: IL-6 and STATTIC Time point: 24 hours

Experiment: MTT Assay

Table format: Grouped	A				B			
	Without NAC				With NAC			
	A:Y1	A:Y2	A:Y3	A:Y4	B:Y1	B:Y2	B:Y3	B:Y4
1 Control	100.000000	100.000000	100.000000	100.000000	100.000000	100.000000	100.000000	100.000000
2 2uM Stattic	28.299120	63.296700	60.173160	63.562280	114.000000	99.014240	98.641000	130.662000
3 4uM Stattic	1.612903	1.538462	1.515152	1.629802	46.941180	70.974810	83.918460	115.679400

Figure4

a. Cell: Huh7 Cytokine: Untreated and IL-6 Time point: 72 hours Experiment:Western Blotting ph-p65 and Total p65(CST), β-Actin(Biobharati)

Control IL-6

Ph-p65

Total-p65

β-Actin

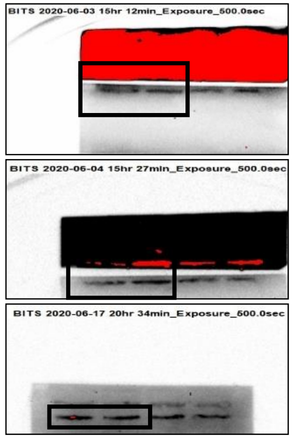

b. Cell: Huh7 Cytokine: IL-6 Time point: 4 hours Experiment: Immunofluorescence Total p65(CST)

Control

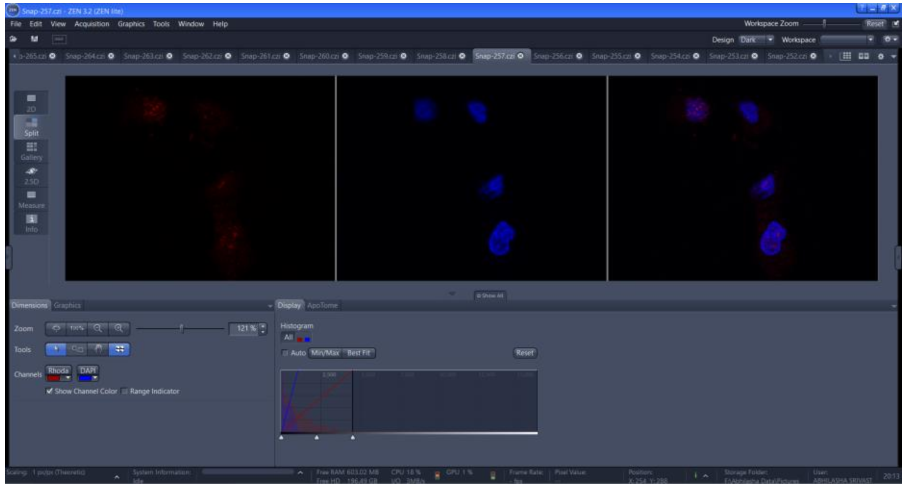

IL-6

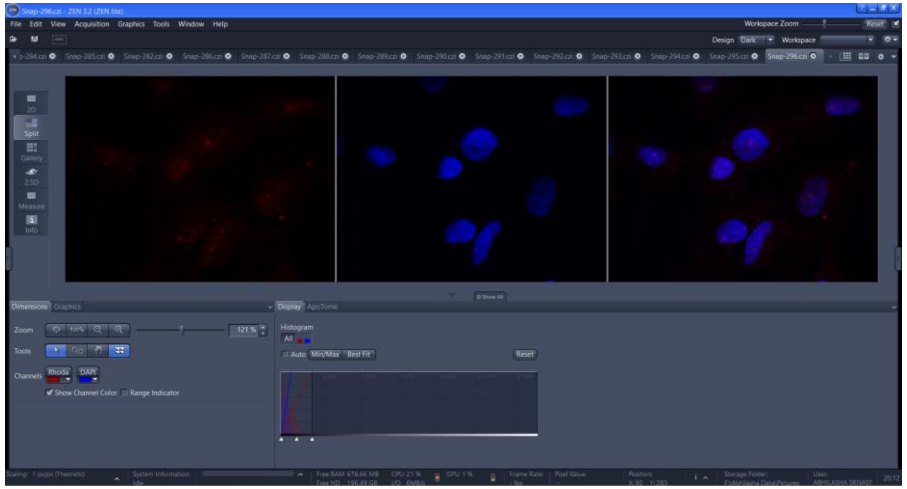

c. Cell: Huh7 Cytokine: IL-6 and p65siRNATime point: 72 hours Experiment:Western Blotting ph-p65 and Total p65(CST), β-Actin(Biobharati)

IL-6	-	+	+	-
NFkB-p65-siRNA	-	-	+	+

ph-p65

Total-p65

β-Actin

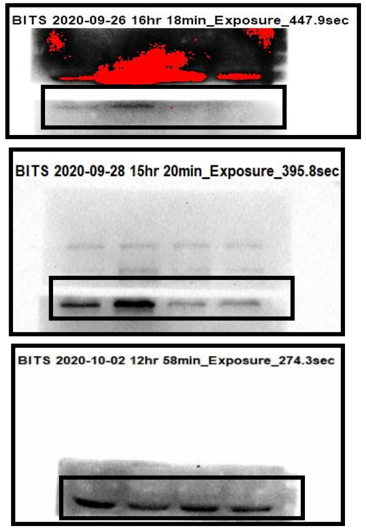

. Cell: Huh7 Cytokine: IL-6 and p65SiRNA Time point: 72hours

Experiment: Flow cytometerAnnexin-PI

Count

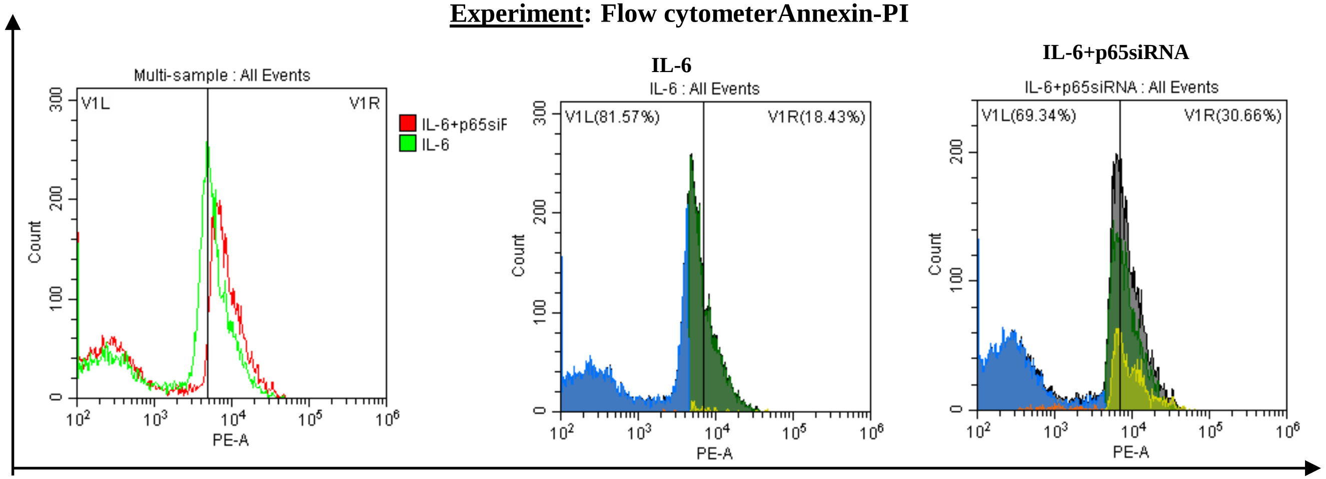

Green Fluorescence/FITC/DCFDA

e. Cell: Huh7 Cytokine: IL-6 and p65siRNATime point: 72 hours Experiment:Western Blotting PCNA(CST), β-Actin(Biobharati)

IL-6	+	+	-
NFkB-p65-siRNA	-	+	+

PCNA

β-Actin

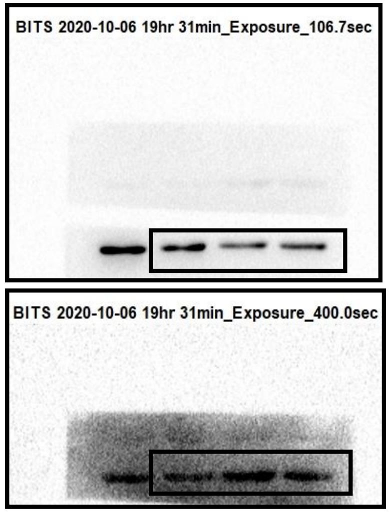

Continued on the next slide

f(i). Cell: Huh7 Cytokine: IL-6 and p65siRNA Time point: 72 hours
Experiment: Western Blotting ph-STAT3, Total STAT3(CST), β-Actin(Biobharati)

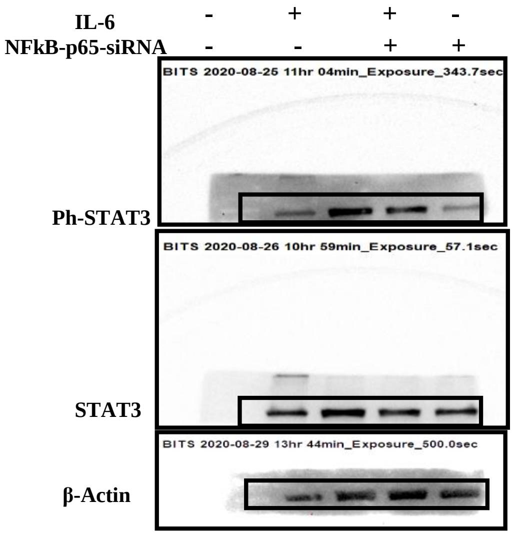

f(ii). Cell: Huh7 Cytokine: IL-6 and STATTIC Time point: 24 hours
Experiment: Western Blotting ph-STAT3, Total STAT3(CST), Total p65 (CST), PCNA (CST) β-Actin(Biobharati)

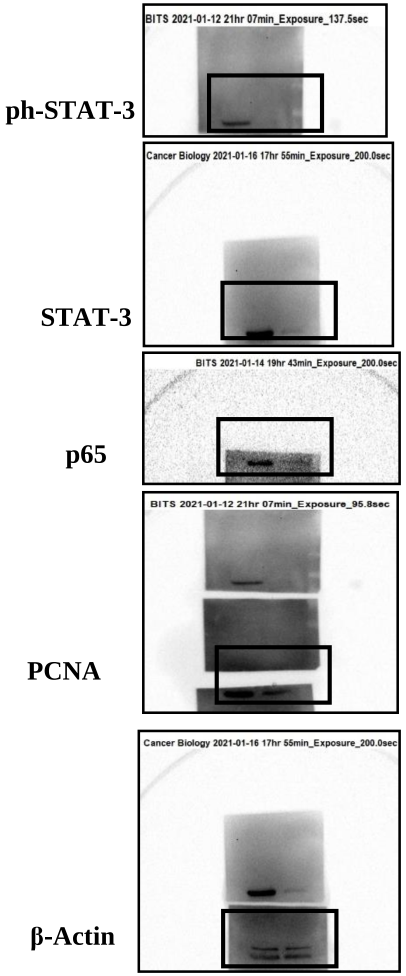

g. Cell: Huh7 Cytokine: IL-6 Time point: 72 hours
Experiment: Western Blotting Total STAT3(CST), Total p65 (CST)

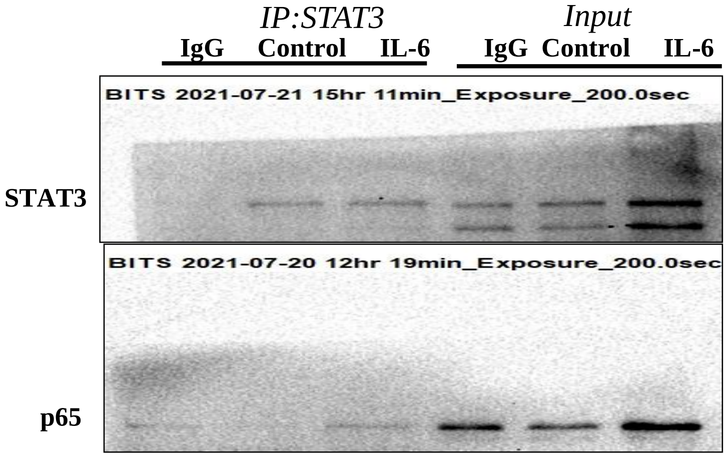

Figure5

b. __Cell__: Huh7 __Cytokine__: TGF-β __Time point__: 72 hours

__Experiment__: RT-PCR:N-Cad

	A	B
	Control	TGF
	Y	Y
1	1	3.668016
2	1	3.771138
3	1	
4	1	1.652901
5	1	2.370186

b. __Cell__: Huh7 __Cytokine__: TGF-β __Time point__: 72 hours

__Experiment__: RT-PCR:Vimentin

	A	B
	Control	TGF
	Y	Y
1	1	3.518596
2	1	1.057018
3	1	1.827663

c. __Cell__: Huh7 __Cytokine__: TGF-β __Time point__: 72 hours

__Experiment__: Western Blotting: N-Cad, Vimentin(CST) β-Actin(Biobharati)

Control TGF-β

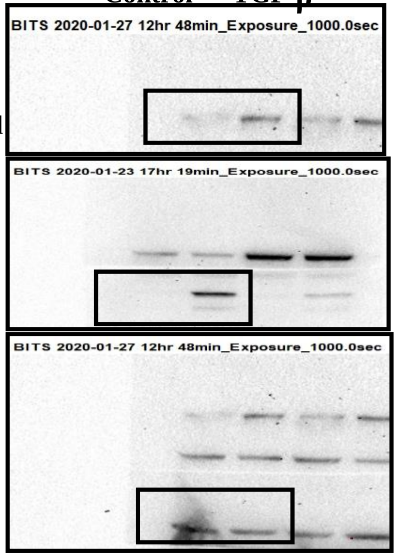

d. __Cell__: Huh7 __Cytokine__: TGF-β __Time point__: 72 hours

__Experiment__: Western Blotting: Smad2/3 complex(CST) β-Actin(Biobharati)

Control TGF-β

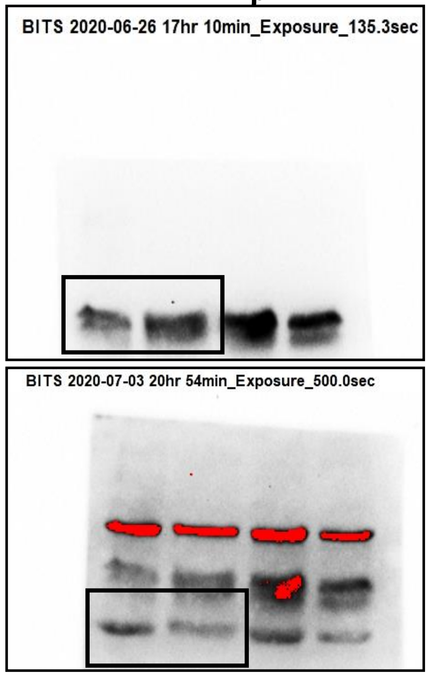

d. __Cell__: Huh7 __Cytokine__: TGF-β __Time point__: 4 hours

__Experiment__: Immunofluorescence: Smad2

	Group A	Group B
	Control	TGF
1	1	4.223708
2	1	5.535046
3	1	3.194241
4	1	1.480861
5	1	1.684239
6	1	3.743441
7	1	2.156761
8	1	5.232372
9	1	2.657021
10	1	1.649624
11	1	2.462889
12	1	4.847771
13	1	2.095157
14	1	1.237235
15	1	3.253985
16	1	2.806858
17	1	2.795482
18	1	2.066888
19	1	1.981937
20	1	2.952034
21	1	5.762712
22	1	7.514896
23	1	3.098539
24	1	4.893908
25	1	2.786067
26	1	2.645927
27	1	2.064920
28	1	2.738991
29	1	5.841393
30	1	2.788859
31	1	5.673574
32	1	3.145868
33	1	2.682249
34	1	2.588275
35	1	5.406887

__Cell__: Huh7 __Cytokine__: TGF-β __Time point__: 4 hours __Experiment__: Immunofluorescence Total SMAD2 (CST)

Control

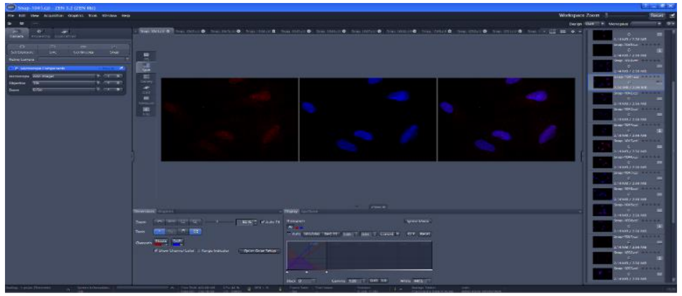

TGF-β

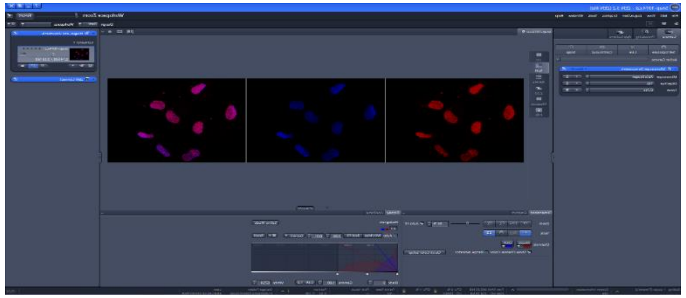

Continued on the next slide

e. <u>Cell</u>: Huh7 <u>Cytokine</u>: TGF-β <u>Time point</u>: 72 hours

<u>Experiment</u>: Western Blotting: N-Cad, Total Smad3(CST) β-Actin(Biobharati)

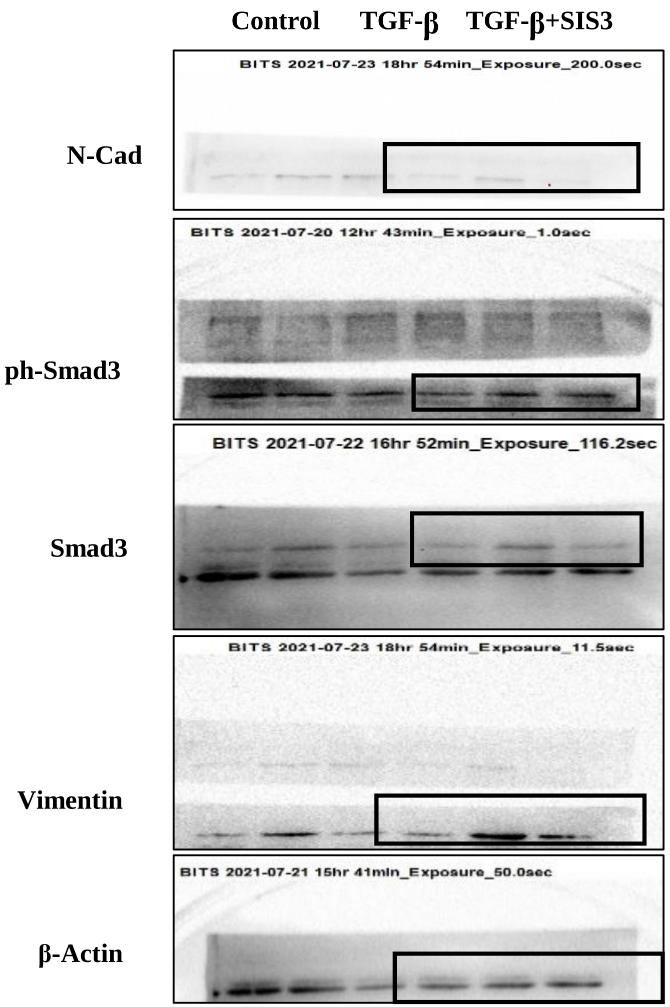

f. <u>Cell</u>: Huh7 <u>Cytokine</u>: TGF-β <u>Time point</u>: 72 hours

<u>Experiment</u>: Western Blotting: N-Cad, Total Smad3(CST) β-Actin(Biobharati)

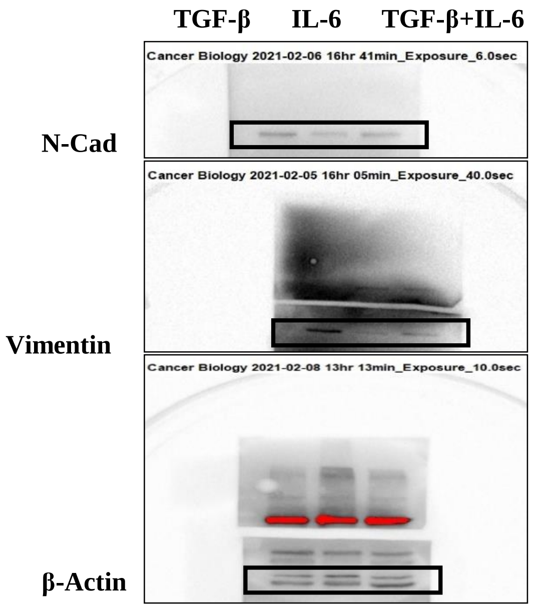

Figure6

b. Cell: Huh7 Cytokine: TGF-β Time point: 72 hours Experiment: MTT assay

	A	B
	Control	TGF
	Y	Y
1	100	38.888890
2	100	29.924240
3	100	24.820140
4	100	71.180560

b. Cell: Huh7 Cytokine: IL-6 and TGF-β Time point: 72 hours Experiment: MTT assay

	Group A	Group B	Group C
	IL-6	TGF-	IL+TGF
1	100.0000	28.22580	30.64515
2	100.0000	25.48388	30.96776
3	100.0000	19.45137	46.79605
4	100.0000	63.46748	39.62847

c. Cell: Huh7 Cytokine: IL-6 and TGF-β Time point: 72 hours Experiment: Cell cycle assay

Table format: Grouped		A				B				C				D			
		Sub G1-Phase				G1-Phase				S-Phase				G2-Phase			
		A:Y1	A:Y2	A:Y3	A:Y4	B:Y1	B:Y2	B:Y3	B:Y4	C:Y1	C:Y2	C:Y3	C:Y4	D:Y1	D:Y2	D:Y3	D:Y4
1	Control	0.44	1.09	4.21	8.71	13.05	22.88	29.88	34.67	73.51	62.92	55.69	47.25	6.28	5.38	2.83	2.68
2	IL-6	1.76	7.80	20.57	29.82	42.90	52.42	44.13	39.55	41.96	30.49	26.55	21.81	2.42	1.52	1.45	1.09
3	TGF	1.04	3.14	7.77	10.91	22.74	31.64	33.93	37.70	64.73	54.15	47.16	39.74	3.76	2.89	1.64	1.87
4	TGF+IL-6	3.15	8.86	18.78	27.49	45.34	52.18	48.46	42.00	37.55	27.54	23.64	22.42	2.08	1.30	1.01	0.77

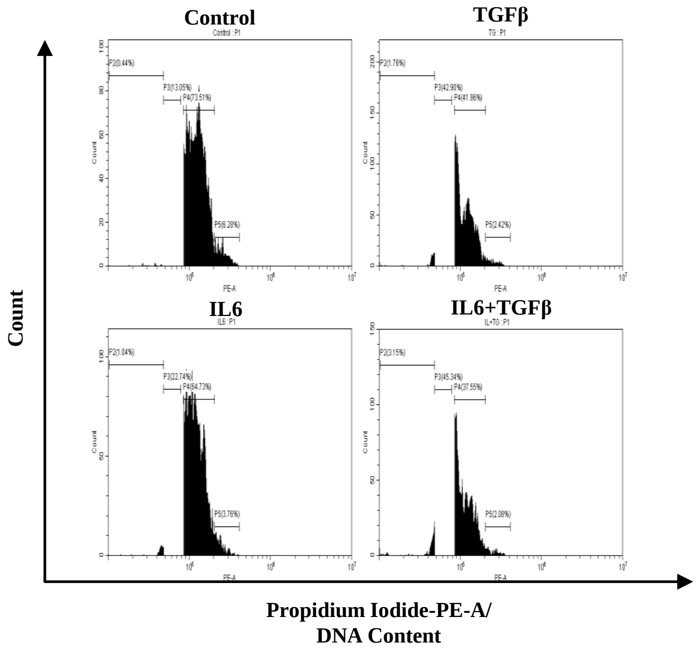

d. Cell: Huh7 Cytokine: IL-6 and TGF-β Time point: 72 hours Experiment: RT-PCR: Ki67

	A	B	C	D
	Control	TGF	IL-6	TGF+IL-6
	Y	Y	Y	Y
1	1.00	4.29		1.46
2	1.00	3.79	52.95	1.49
3	1.00	3.45	38.97	1.93
4	1.00	3.79	52.95	1.49

d. Cell: Huh7 Cytokine: IL-6 and TGF-β Time point: 72 hours Experiment: RT-PCR: PCNA

	A	B	C	D
	Control	TGF	IL-6	TGF+IL-6
	Y	Y	Y	Y
1	1.00	2.88	224.71	0.88
2	1.00	3.29	376.10	0.68
3	1.00	2.88	224.71	0.88
4	1.00	3.29	376.10	0.68

Figure7

a. Cell: Huh7 Cytokine: IL-6 and TGF-β Time point: 72 hours Experiment: Western Blotting phospho-p65Total p65(CST), β-Actin(Biobharati)

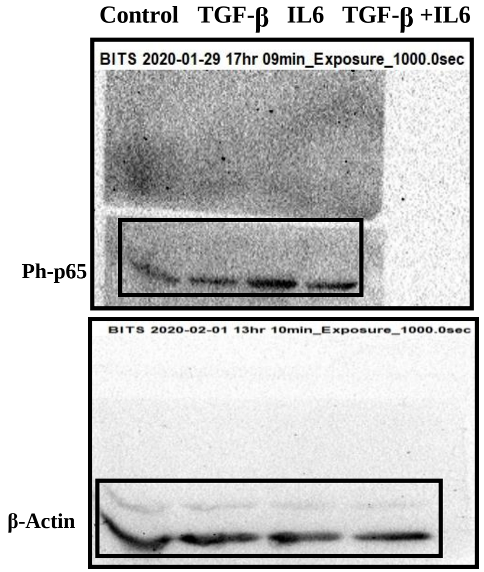

b.Cell: Huh7 Cytokine: TGF-β and IL-6 Time point: 72 hours Experiment: Immunofluorescence Total p65(CST)

IL-6

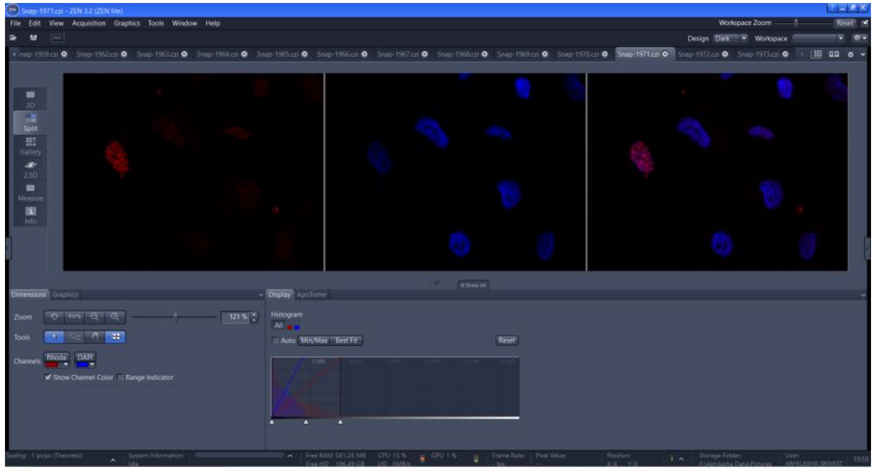

TGF-β

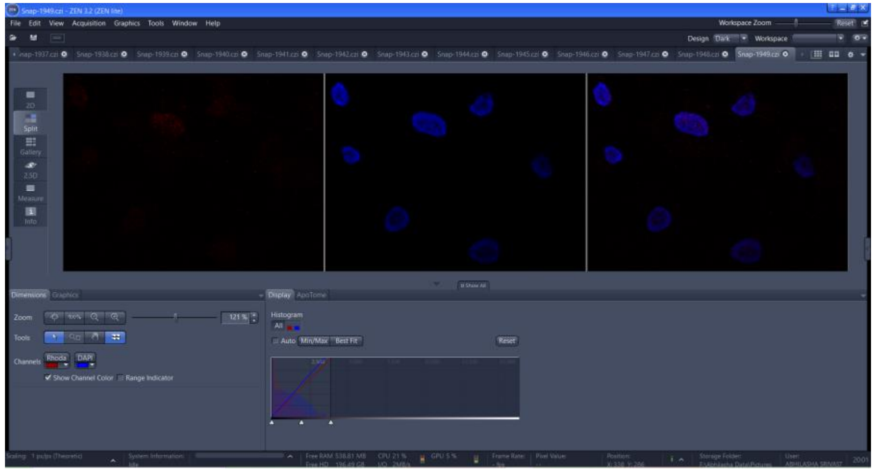

TGF-β+IL-6

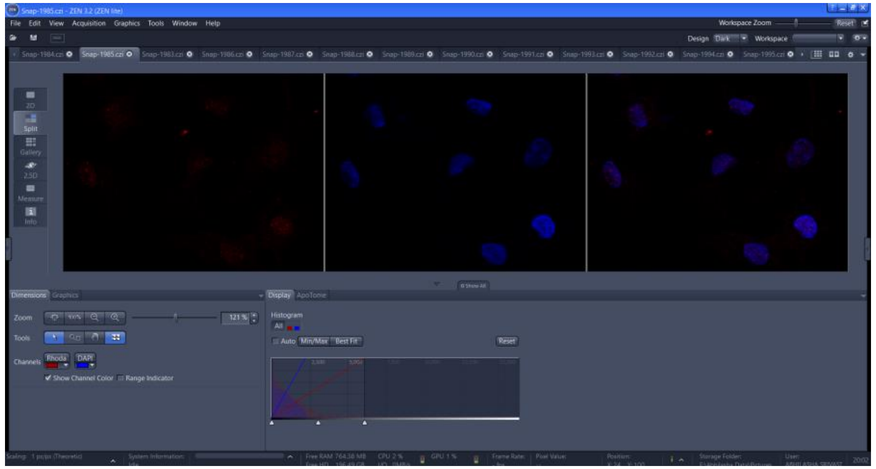

Continued on the next slide

c. Cell: Huh7 Cytokine: IL-6 and TGF-β Time point: 72 hours Experiment: RT-PCR: IL-6R

	A	B	C	D
	Control	TGF	IL-6	TGF+IL-6
	Y	Y	Y	Y
1	1.00	3.35	207.72	1.75
2	1.00	5.34	313.12	1.07
3	1.00	0.38	260.42	3.75
4	1.00	0.38		0.43

d. Cell: Huh7 Cytokine: IL-6 and TGF-β Time point: 72 hours Experiment: Western Blotting :PCNA (CST), β-Actin(Biobharati)

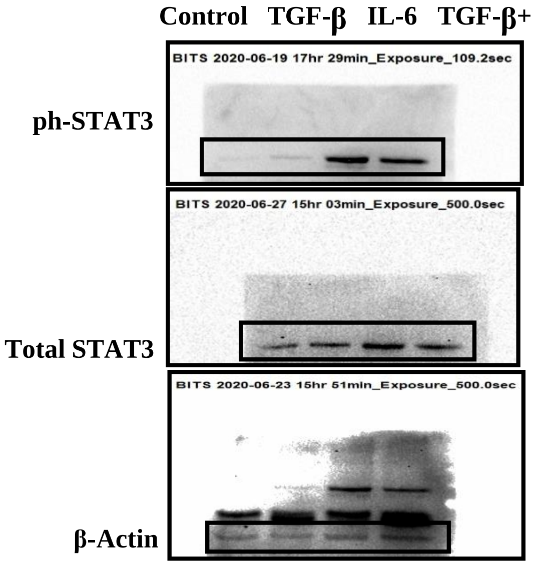

e. Cell: Huh7 Cytokine: IL-6 and TGF-β Time point: 72 hours Experiment: Immunofluorescence: STAT3

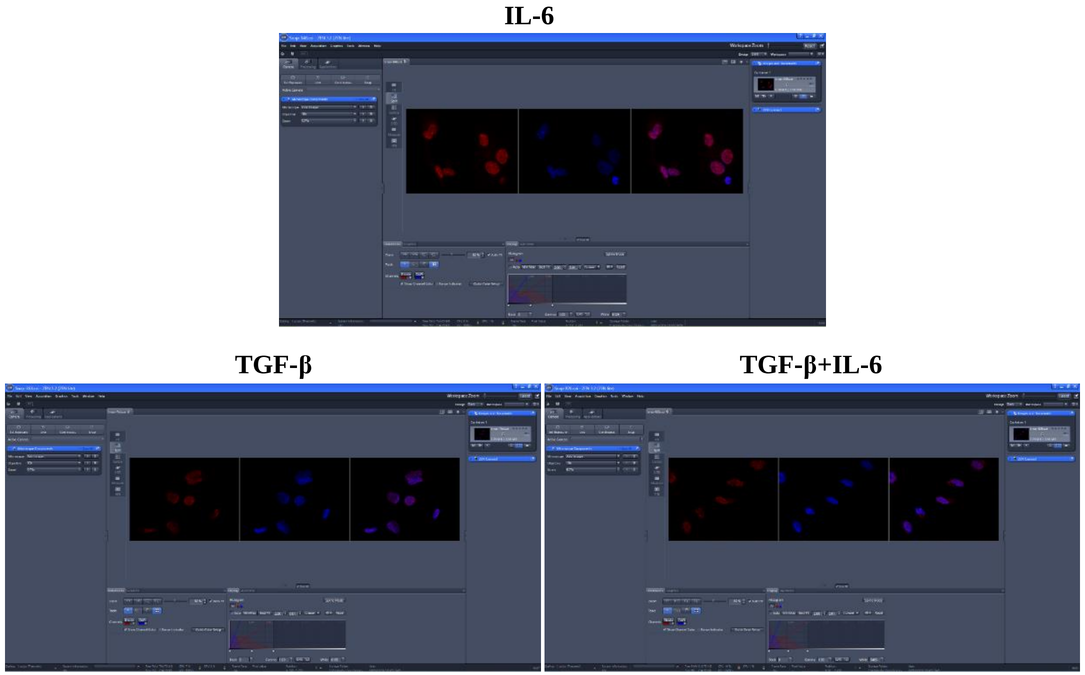

	A	B	C	D
	Control	TGF	IL-6	TGF+IL-6
	Y	Y	Y	Y
1	1	1.975375	4.684109	2.182634
2	1	1.880175	3.353578	1.370030
3	1	4.657330	7.217819	2.015197
4	1	0.856154	6.118980	1.293359
5	1	0.931759	5.343215	1.764860
6	1	0.981600	6.940600	4.438000
7	1	0.781250	4.486094	3.281250

F. Cell: Huh7 Cytokine: IL-6 and TGF-β Time point: 72 hours Experiment: Immunofluorescence: Smad2

	A	B	C	D
	Control	TGF	IL-6	TGF+IL-6
	Y	Y	Y	Y
1	1	1.267860	1.067980	1.638743
2	1	0.648463	0.597625	1.258630
3	1	1.321554	0.583606	1.445862
4	1	1.016655	1.014766	1.067994
5	1	2.682135	0.881523	1.852116
6	1	2.394000	1.292250	2.834750
7	1	1.229000	1.182000	2.561200
8	1	2.037125	0.648250	0.777500

F. Cell: Huh7 Cytokine: IL-6 and TGF-β Time point: 72 hours Experiment:Western Blotting Smad2/3 Complex(CST), β-Actin(Biobharati)

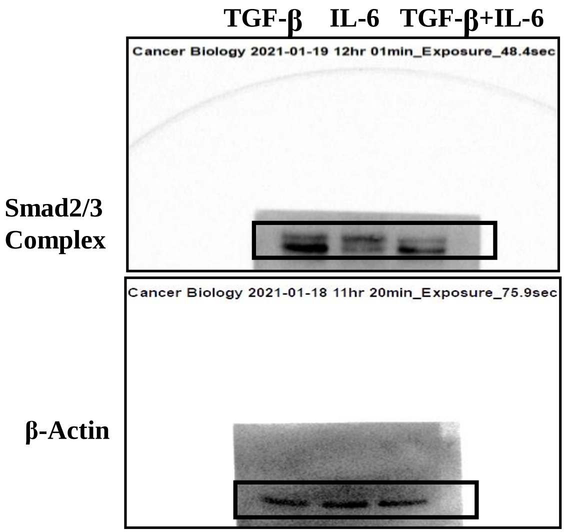

Continued on the next slide

f. Cell: Huh7   Cytokine: TGF-β and IL-6 Time point: 4 hours   Experiment: Immunofluorescence Total SMAD2 (CST)

TGF-β

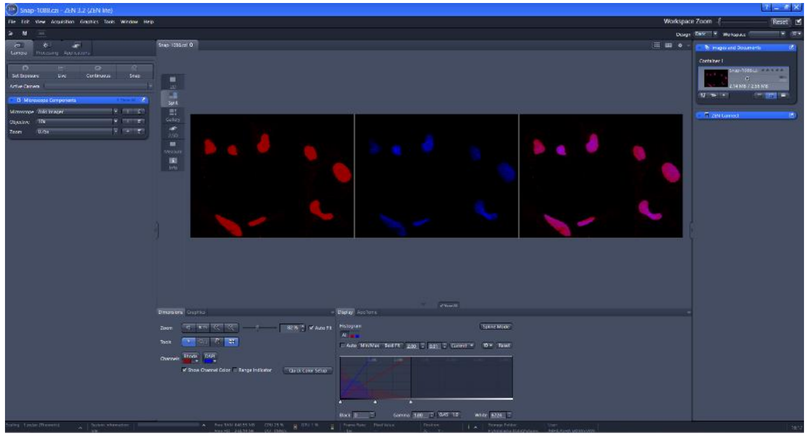

IL-6

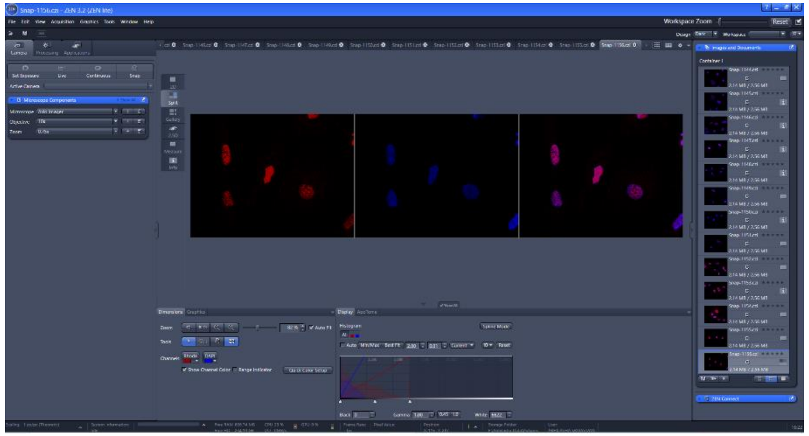

TGF-β+IL-6

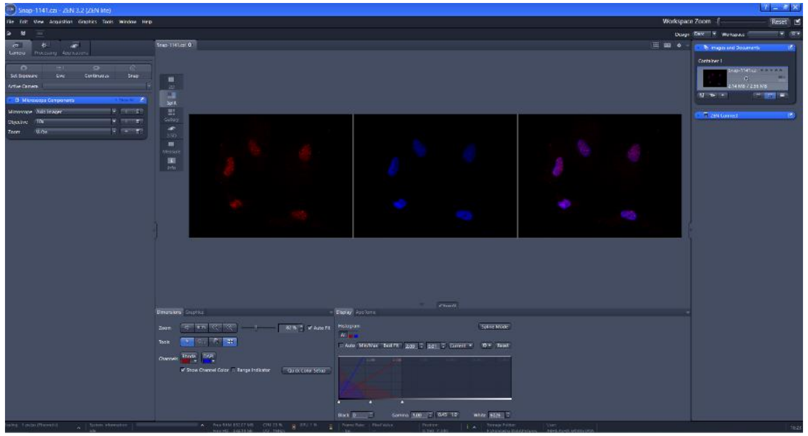

Figure8

a. Cell: Huh7 Cytokine: IL-6 Time point: 72 hours Experiment:Western Blotting H3K4me3(CST), β-Actin(Biobharati)

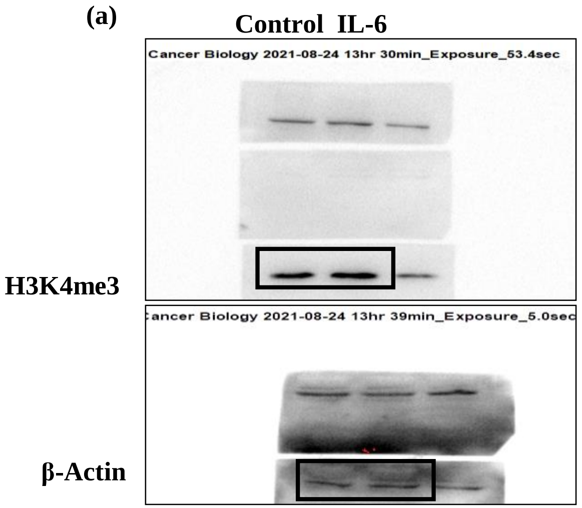

a. Cell: Huh7 Cytokine: IL-6 Time point: 72 hours Experiment:Western Blotting H3K27me3(CST), β-Actin(Biobharati)

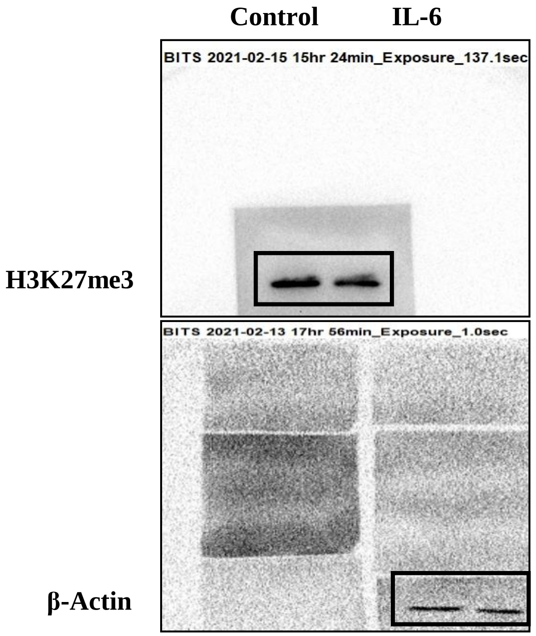

b. Cell: Huh7 Cytokine: IL-6 Time point: 72 hours Experiment:Western Blotting H3K4me3(CST), β-Actin(Biobharati)

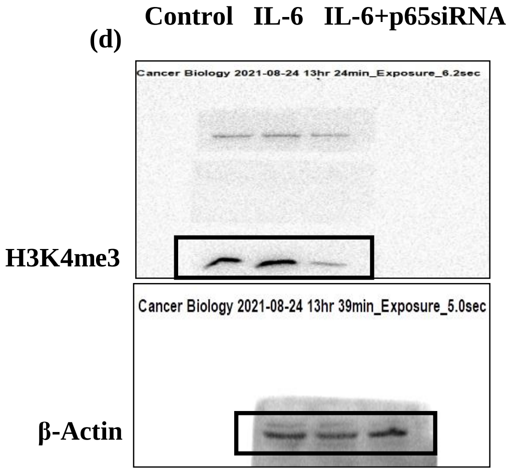

f. Cell: Huh7 Cytokine: TGF-β and IL-6 Time point: 4 hours Experiment: Immunofluorescence EZH2 (CST)

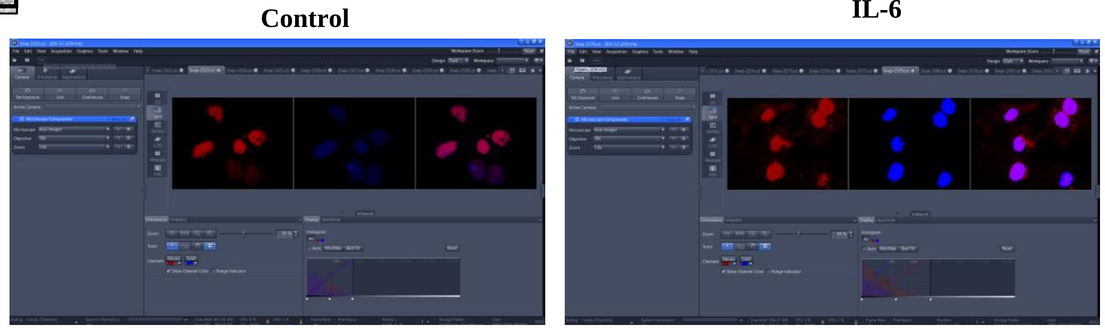

c. Cell: Huh7 Cytokine: TGF-β Time point: 72 hours Experiment:Western Blotting H3K27me3(CST), β-Actin(Biobharati)

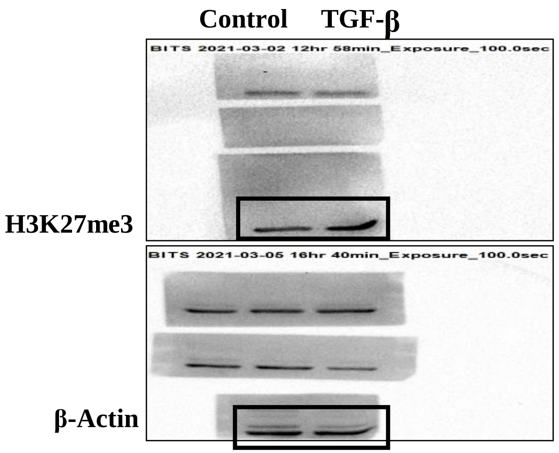

d. Cell: Huh7 Cytokine: IL-6 Time point: 72 hours Experiment:Western Blotting EZH2(CST), β-Actin(Biobharati)

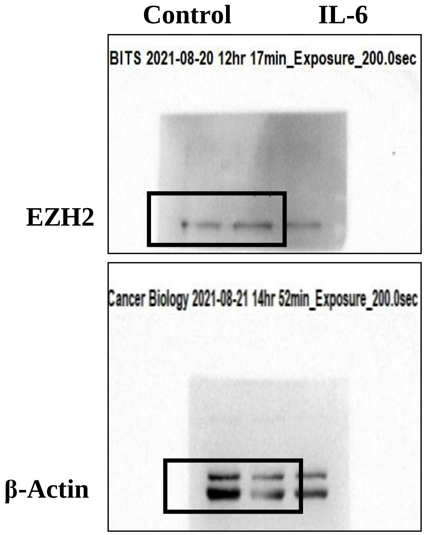

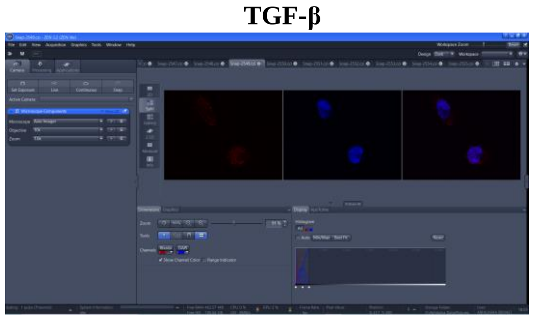

f. Cell: Huh7 Cytokine: IL-6 Time point: 72 hours

Experiment: MTT Assay

Table format: Grouped		Group A				Group B			
		Control				IL-6			
	×	A:1	A:2	A:3	A:4	B:1	B:2	B:3	B:4
1	Title	100.00000	100.00000	100.0000	100.0000	104.47760	113.24200	127.52290	135.78950
2	Title	135.82090	136.07310	138.9908	142.6316	133.33330	123.28770	114.67890	131.05260
3	Title	108.45770	111.41550	119.2661	115.2632	118.40800	111.41550	111.92660	123.15790
4	Title	87.06468	91.78082	101.3761	100.0000	81.59204	78.08219	69.72477	58.94737

RT-PCR raw data for IL-6R,gp-130 and B-Actin

IL6R-mRNA
Date: 23.10.2020

Data Analysis - admin_2020-10-23 09-06-27_BR001889.pcrd

File View Settings Export Tools

Well Group: All Wells | Analysis Mode: Fluorophore | View/Edit Plate...

Quantification | Quantification Data | Melt Curve | Melt Curve Data | Gene Expression | End Point | Custom Data View | QC | Run Information

Rows: 2 Columns: 3 Load a Preset View: <Select a preset View> Save as Preset... Manage Presets...

Amplification Chart

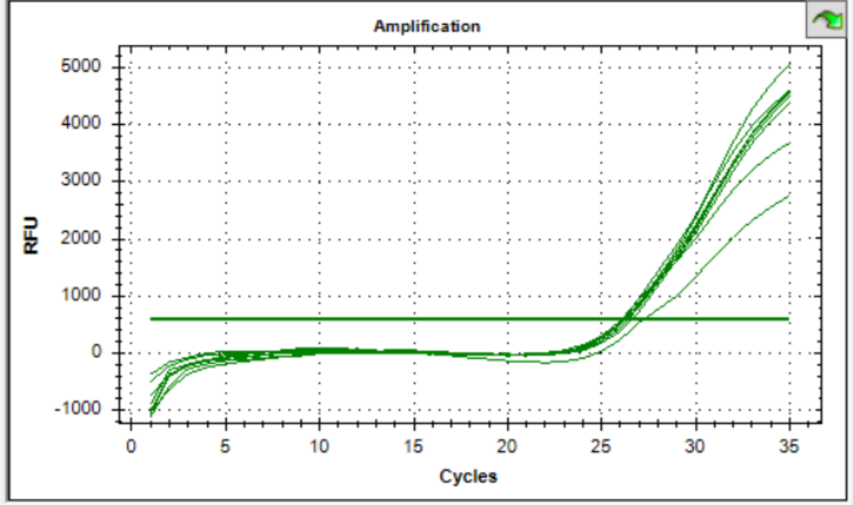

Melt Curve Chart

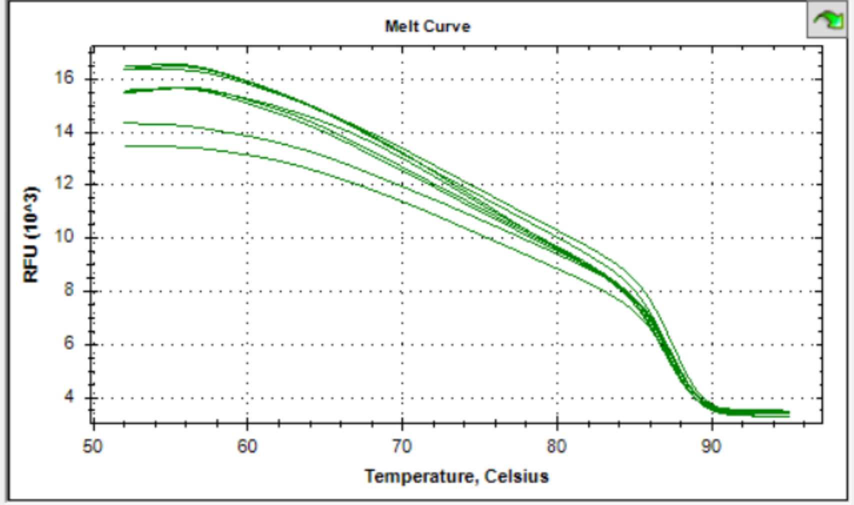

Melt Peaks Chart

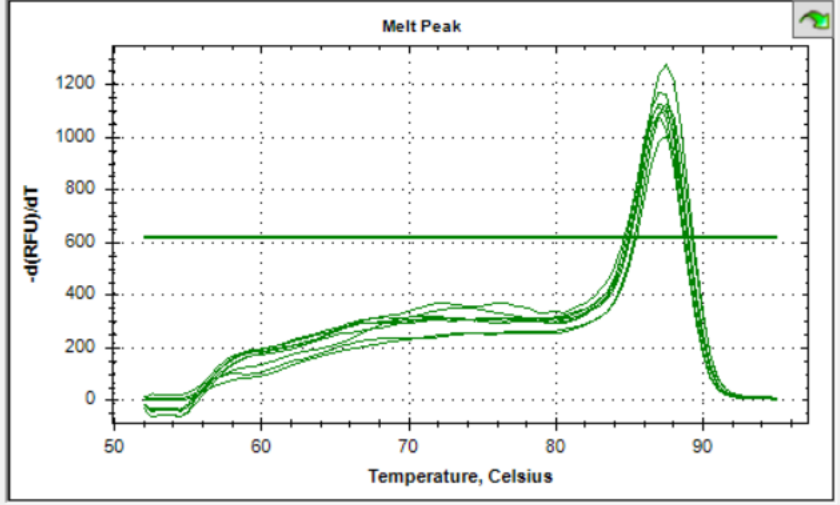

Quantification Summary Results Grid

Well	Fluor	Target	Content	Sample	Cq
D01	SYBR	IL6R	Unkn	Control	26.29
D02	SYBR	IL6R	Unkn	TG	26.45
D03	SYBR	IL6R	Unkn	IL	26.28
D04	SYBR	IL6R	Unkn	IL+TG	26.63
D05	SYBR	IL6R	Unkn	Control	26.40
D06	SYBR	IL6R	Unkn	TG	26.19
D07	SYBR	IL6R	Unkn	IL	26.11
D08	SYBR	IL6R	Unkn	IL+TG	27.31

Melt Curve Summary Results Grid

Well	Fluor	Target	Content	Sample	Melt Temp
D01	SYBR	IL6R	Unkn	Control	87.50
D02	SYBR	IL6R	Unkn	TG	87.00
D03	SYBR	IL6R	Unkn	IL	87.00
D04	SYBR	IL6R	Unkn	IL+TG	87.00
D05	SYBR	IL6R	Unkn	Control	87.00
D06	SYBR	IL6R	Unkn	TG	87.50
D07	SYBR	IL6R	Unkn	IL	87.50
D08	SYBR	IL6R	Unkn	IL+TG	87.50

Well Selector

	1	2	3	4	5	6	7	8	9	10	11	12
A	Unk	Unk	Unk	Unk	Unk	Unk	Unk	Unk				
B	Unk	Unk	Unk	Unk	Unk	Unk	Unk	Unk				
C	Unk	Unk	Unk	Unk	Unk	Unk	Unk	Unk				
D	Unk	Unk	Unk	Unk	Unk	Unk	Unk	Unk				
E	Unk	Unk	Unk	Unk	Unk	Unk	Unk	Unk				
F	Unk	Unk	Unk	Unk	Unk	Unk	Unk	Unk				
G	Unk	Unk	Unk	Unk	Unk	Unk	Unk	Unk				
H	Unk	Unk	Unk	Unk	Unk	Unk	Unk	Unk				

Completed | Scan Mode: All Channels | Plate Type: BR Clear | Analysis Mode: Baseline Subtracted Curve Fit

gp130-mRNA
Date: 23.10.2020

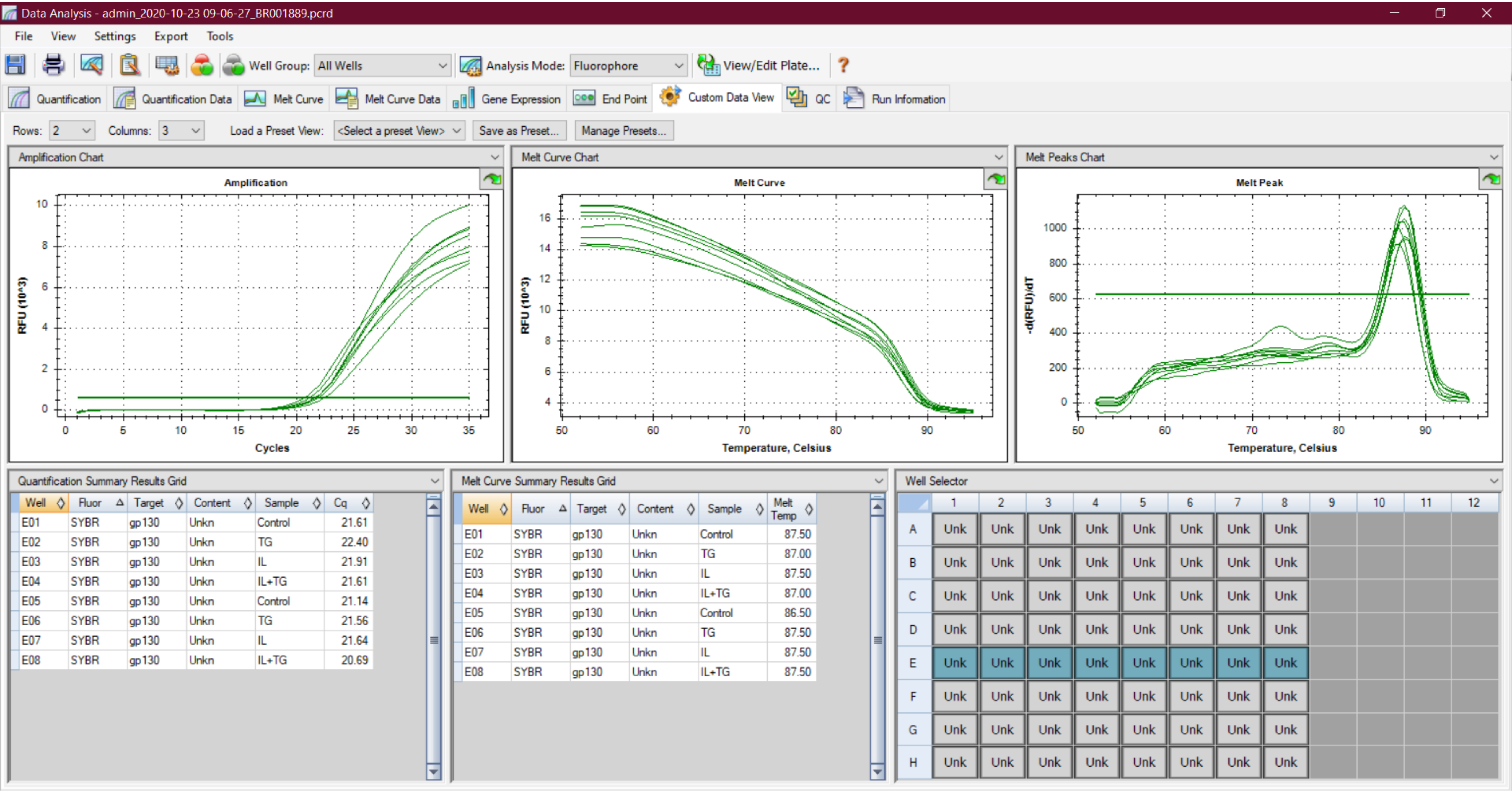

Quantification Summary Results Grid

Well	Fluor	Target	Content	Sample	Cq
E01	SYBR	gp130	Unkn	Control	21.61
E02	SYBR	gp130	Unkn	TG	22.40
E03	SYBR	gp130	Unkn	IL	21.91
E04	SYBR	gp130	Unkn	IL+TG	21.61
E05	SYBR	gp130	Unkn	Control	21.14
E06	SYBR	gp130	Unkn	TG	21.56
E07	SYBR	gp130	Unkn	IL	21.64
E08	SYBR	gp130	Unkn	IL+TG	20.69

Melt Curve Summary Results Grid

Well	Fluor	Target	Content	Sample	Melt Temp
E01	SYBR	gp130	Unkn	Control	87.50
E02	SYBR	gp130	Unkn	TG	87.00
E03	SYBR	gp130	Unkn	IL	87.50
E04	SYBR	gp130	Unkn	IL+TG	87.00
E05	SYBR	gp130	Unkn	Control	86.50
E06	SYBR	gp130	Unkn	TG	87.50
E07	SYBR	gp130	Unkn	IL	87.50
E08	SYBR	gp130	Unkn	IL+TG	87.50

Well Selector

	1	2	3	4	5	6	7	8	9	10	11	12
A	Unk	Unk	Unk	Unk	Unk	Unk	Unk	Unk				
B	Unk	Unk	Unk	Unk	Unk	Unk	Unk	Unk				
C	Unk	Unk	Unk	Unk	Unk	Unk	Unk	Unk				
D	Unk	Unk	Unk	Unk	Unk	Unk	Unk	Unk				
E	Unk	Unk	Unk	Unk	Unk	Unk	Unk	Unk				
F	Unk	Unk	Unk	Unk	Unk	Unk	Unk	Unk				
G	Unk	Unk	Unk	Unk	Unk	Unk	Unk	Unk				
H	Unk	Unk	Unk	Unk	Unk	Unk	Unk	Unk				

B-Actin-mRNA
Date: 23.10.2020

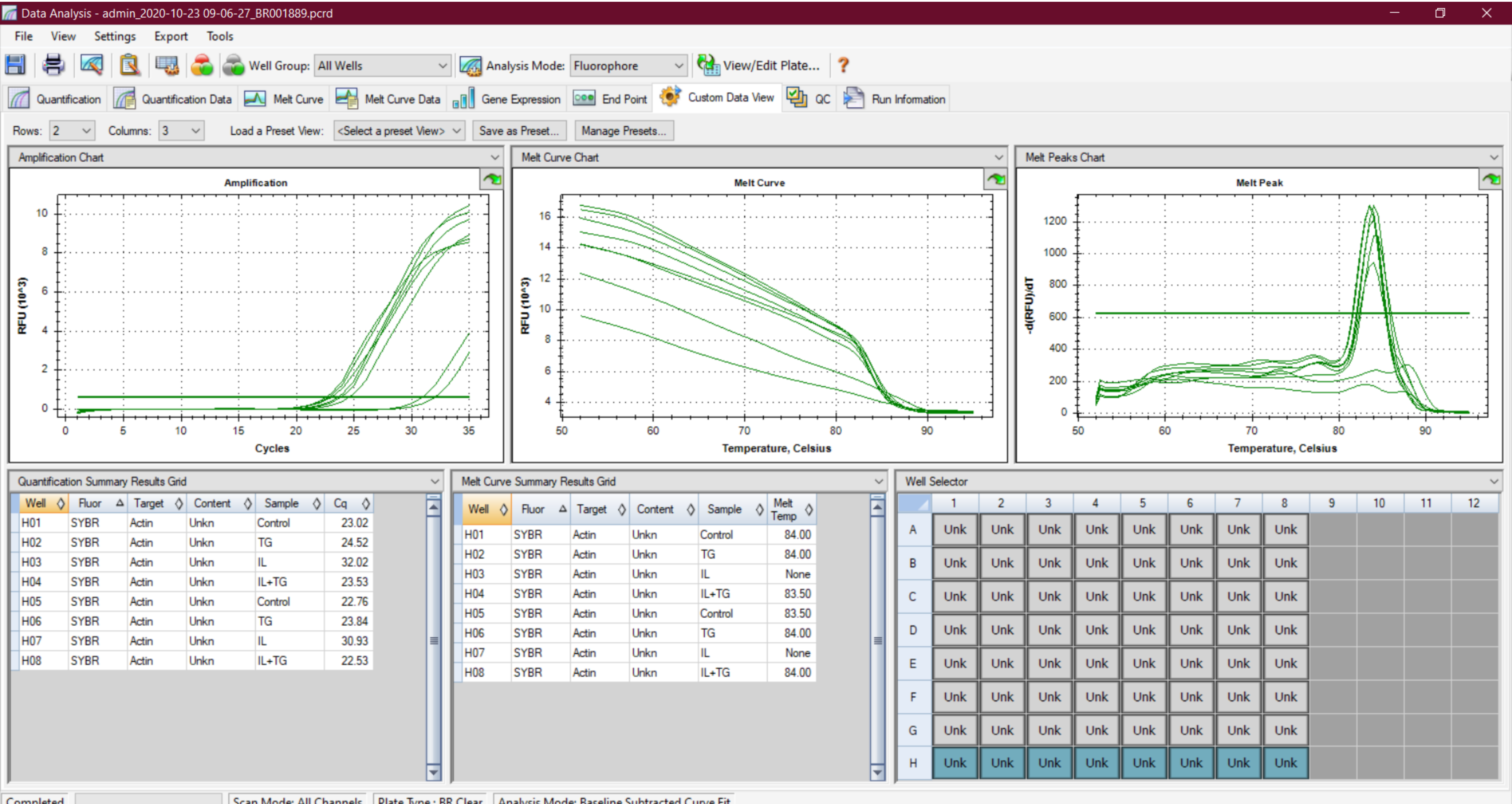

Well	Fluor	Target	Content	Sample	Cq
H01	SYBR	Actin	Unkn	Control	23.02
H02	SYBR	Actin	Unkn	TG	24.52
H03	SYBR	Actin	Unkn	IL	32.02
H04	SYBR	Actin	Unkn	IL+TG	23.53
H05	SYBR	Actin	Unkn	Control	22.76
H06	SYBR	Actin	Unkn	TG	23.84
H07	SYBR	Actin	Unkn	IL	30.93
H08	SYBR	Actin	Unkn	IL+TG	22.53

Well	Fluor	Target	Content	Sample	Melt Temp
H01	SYBR	Actin	Unkn	Control	84.00
H02	SYBR	Actin	Unkn	TG	84.00
H03	SYBR	Actin	Unkn	IL	None
H04	SYBR	Actin	Unkn	IL+TG	83.50
H05	SYBR	Actin	Unkn	Control	83.50
H06	SYBR	Actin	Unkn	TG	84.00
H07	SYBR	Actin	Unkn	IL	None
H08	SYBR	Actin	Unkn	IL+TG	84.00

	1	2	3	4	5	6	7	8	9	10	11	12
A	Unk	Unk	Unk	Unk	Unk	Unk	Unk	Unk				
B	Unk	Unk	Unk	Unk	Unk	Unk	Unk	Unk				
C	Unk	Unk	Unk	Unk	Unk	Unk	Unk	Unk				
D	Unk	Unk	Unk	Unk	Unk	Unk	Unk	Unk				
E	Unk	Unk	Unk	Unk	Unk	Unk	Unk	Unk				
F	Unk	Unk	Unk	Unk	Unk	Unk	Unk	Unk				
G	Unk	Unk	Unk	Unk	Unk	Unk	Unk	Unk				
H	Unk	Unk	Unk	Unk	Unk	Unk	Unk	Unk				

RT-PCR raw data for Ki67, PCNA and B-Actin

PCNA-mRNA
Date: 22.10.2020

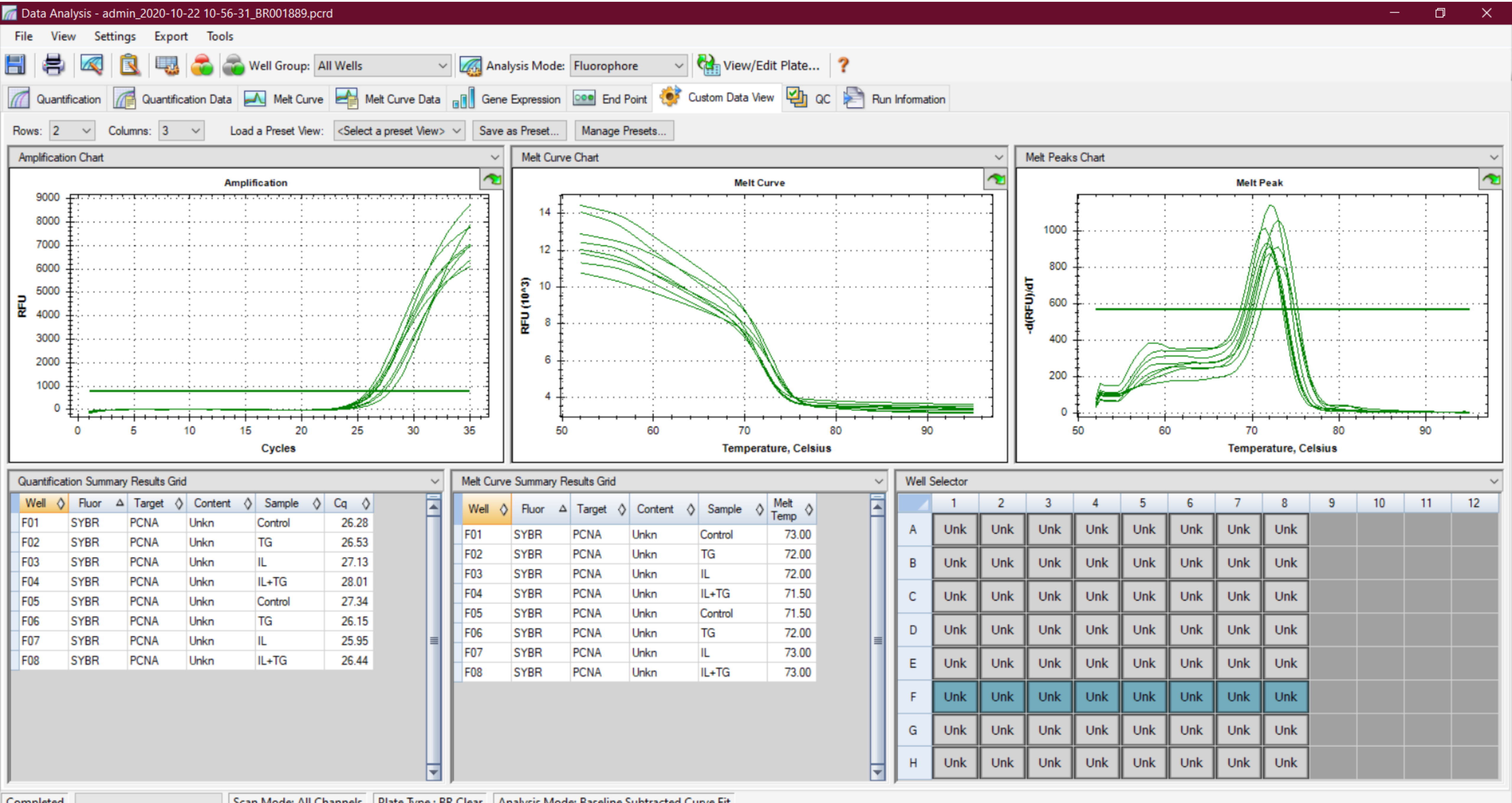

Quantification Summary Results Grid

Well	Fluor	Target	Content	Sample	Cq
F01	SYBR	PCNA	Unkn	Control	26.28
F02	SYBR	PCNA	Unkn	TG	26.53
F03	SYBR	PCNA	Unkn	IL	27.13
F04	SYBR	PCNA	Unkn	IL+TG	28.01
F05	SYBR	PCNA	Unkn	Control	27.34
F06	SYBR	PCNA	Unkn	TG	26.15
F07	SYBR	PCNA	Unkn	IL	25.95
F08	SYBR	PCNA	Unkn	IL+TG	26.44

Melt Curve Summary Results Grid

Well	Fluor	Target	Content	Sample	Melt Temp
F01	SYBR	PCNA	Unkn	Control	73.00
F02	SYBR	PCNA	Unkn	TG	72.00
F03	SYBR	PCNA	Unkn	IL	72.00
F04	SYBR	PCNA	Unkn	IL+TG	71.50
F05	SYBR	PCNA	Unkn	Control	71.50
F06	SYBR	PCNA	Unkn	TG	72.00
F07	SYBR	PCNA	Unkn	IL	73.00
F08	SYBR	PCNA	Unkn	IL+TG	73.00

Ki67-mRNA
Date: 22.10.2020

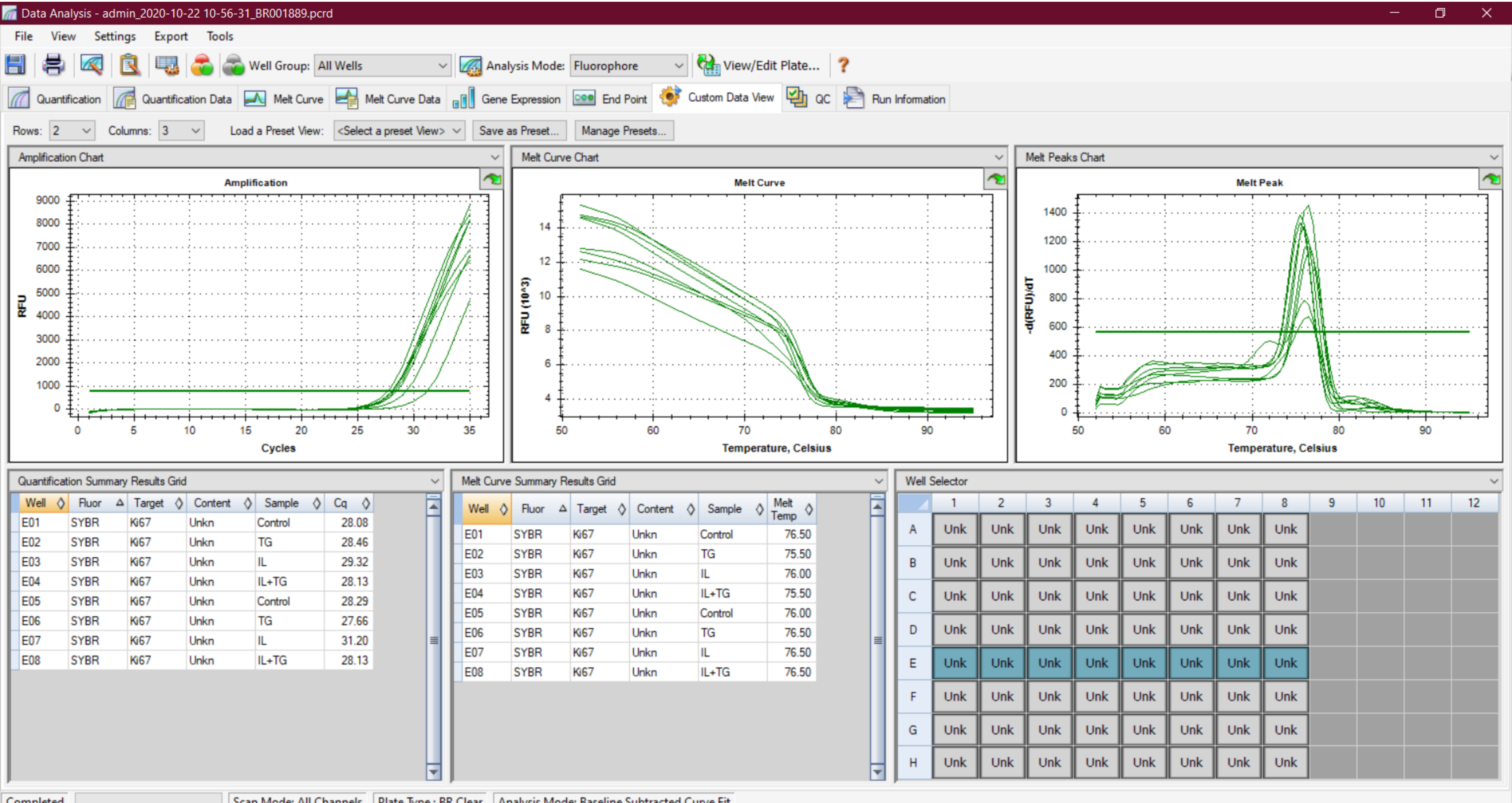

B-Actin-mRNA
Date: 22.10.2020

Data Analysis - admin_2020-10-22 10-56-31_BR001889.pcrd

File View Settings Export Tools

Well Group: All Wells | Analysis Mode: Fluorophore | View/Edit Plate...

Quantification | Quantification Data | Melt Curve | Melt Curve Data | Gene Expression | End Point | Custom Data View | QC | Run Information

Rows: 2 Columns: 3 Load a Preset View: <Select a preset View> Save as Preset... Manage Presets...

Amplification Chart

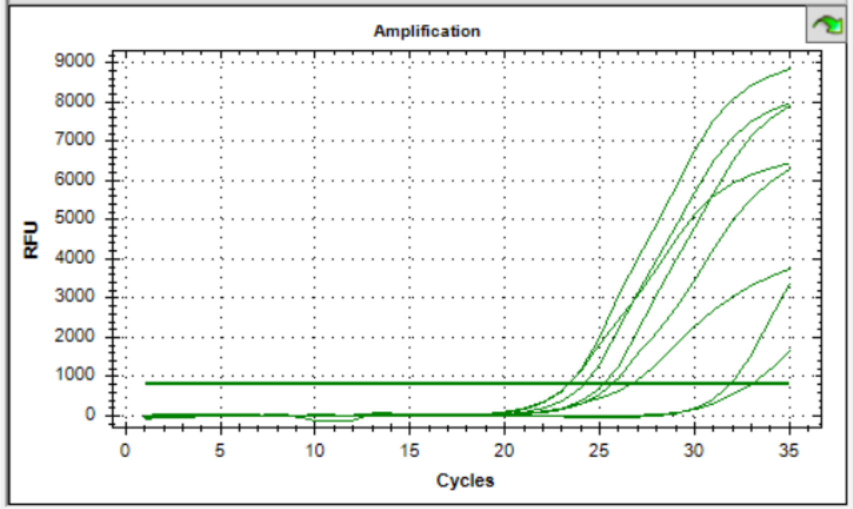

Melt Curve Chart

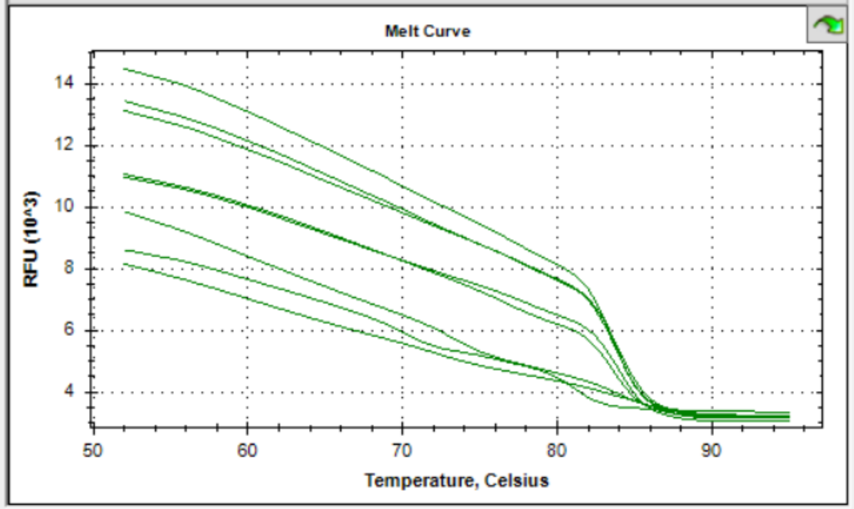

Melt Peaks Chart

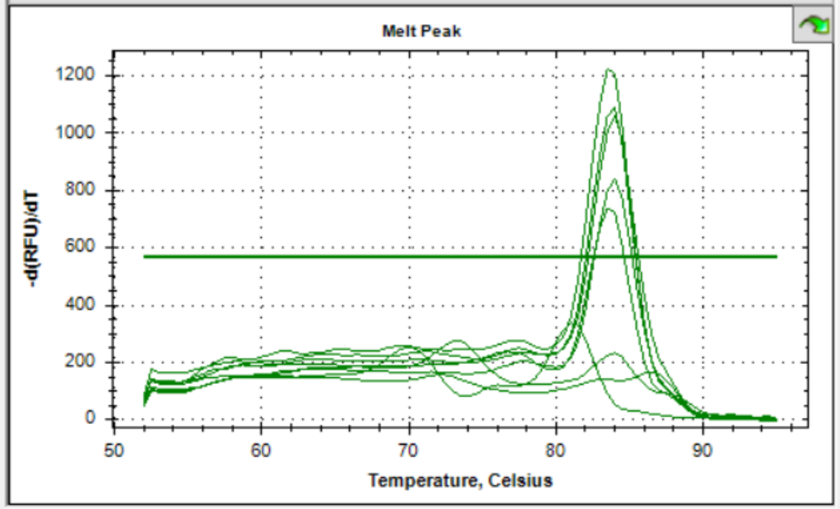

Quantification Summary Results Grid

Well	Fluor	Target	Content	Sample	Cq
B01	SYBR	Actin	Unkn	Control	23.34
B02	SYBR	Actin	Unkn	TG	25.19
B03	SYBR	Actin	Unkn	IL	31.86
B04	SYBR	Actin	Unkn	IL+TG	24.16
B05	SYBR	Actin	Unkn	Control	23.34
B06	SYBR	Actin	Unkn	TG	25.62
B07	SYBR	Actin	Unkn	IL	33.01
B08	SYBR	Actin	Unkn	IL+TG	26.63

Melt Curve Summary Results Grid

Well	Fluor	Target	Content	Sample	Melt Temp
B01	SYBR	Actin	Unkn	Control	83.50
B02	SYBR	Actin	Unkn	TG	84.00
B03	SYBR	Actin	Unkn	IL	None
B04	SYBR	Actin	Unkn	IL+TG	84.00
B05	SYBR	Actin	Unkn	Control	84.00
B06	SYBR	Actin	Unkn	TG	83.50
B07	SYBR	Actin	Unkn	IL	None
B08	SYBR	Actin	Unkn	IL+TG	None

Well Selector

	1	2	3	4	5	6	7	8	9	10	11	12
A	Unk	Unk	Unk	Unk	Unk	Unk	Unk	Unk				
B	Unk	Unk	Unk	Unk	Unk	Unk	Unk	Unk				
C	Unk	Unk	Unk	Unk	Unk	Unk	Unk	Unk				
D	Unk	Unk	Unk	Unk	Unk	Unk	Unk	Unk				
E	Unk	Unk	Unk	Unk	Unk	Unk	Unk	Unk				
F	Unk	Unk	Unk	Unk	Unk	Unk	Unk	Unk				
G	Unk	Unk	Unk	Unk	Unk	Unk	Unk	Unk				
H	Unk	Unk	Unk	Unk	Unk	Unk	Unk	Unk				

Completed | Scan Mode: All Channels | Plate Type: BR Clear | Analysis Mode: Baseline Subtracted Curve Fit

RT-PCR raw data for N-Cad, Vimentin, TGFR and B-Actin

N-Cad-mRNA
Date: 19.10.2020

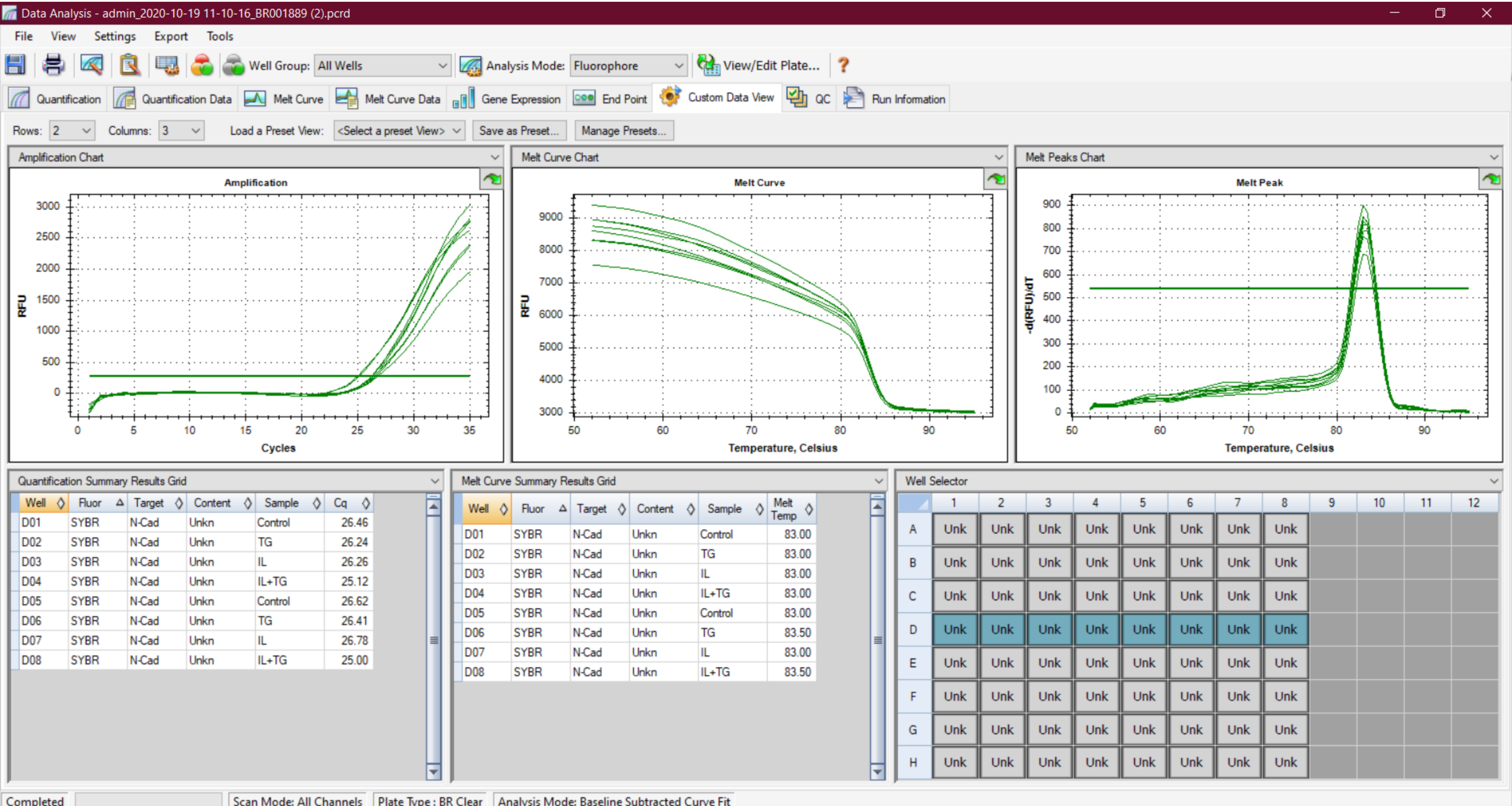

Quantification Summary Results Grid

Well	Fluor	Target	Content	Sample	Cq
D01	SYBR	N-Cad	Unkn	Control	26.46
D02	SYBR	N-Cad	Unkn	TG	26.24
D03	SYBR	N-Cad	Unkn	IL	26.26
D04	SYBR	N-Cad	Unkn	IL+TG	25.12
D05	SYBR	N-Cad	Unkn	Control	26.62
D06	SYBR	N-Cad	Unkn	TG	26.41
D07	SYBR	N-Cad	Unkn	IL	26.78
D08	SYBR	N-Cad	Unkn	IL+TG	25.00

Melt Curve Summary Results Grid

Well	Fluor	Target	Content	Sample	Melt Temp
D01	SYBR	N-Cad	Unkn	Control	83.00
D02	SYBR	N-Cad	Unkn	TG	83.00
D03	SYBR	N-Cad	Unkn	IL	83.00
D04	SYBR	N-Cad	Unkn	IL+TG	83.00
D05	SYBR	N-Cad	Unkn	Control	83.00
D06	SYBR	N-Cad	Unkn	TG	83.50
D07	SYBR	N-Cad	Unkn	IL	83.00
D08	SYBR	N-Cad	Unkn	IL+TG	83.50

Vimentin-mRNA
Date: 19.10.2020

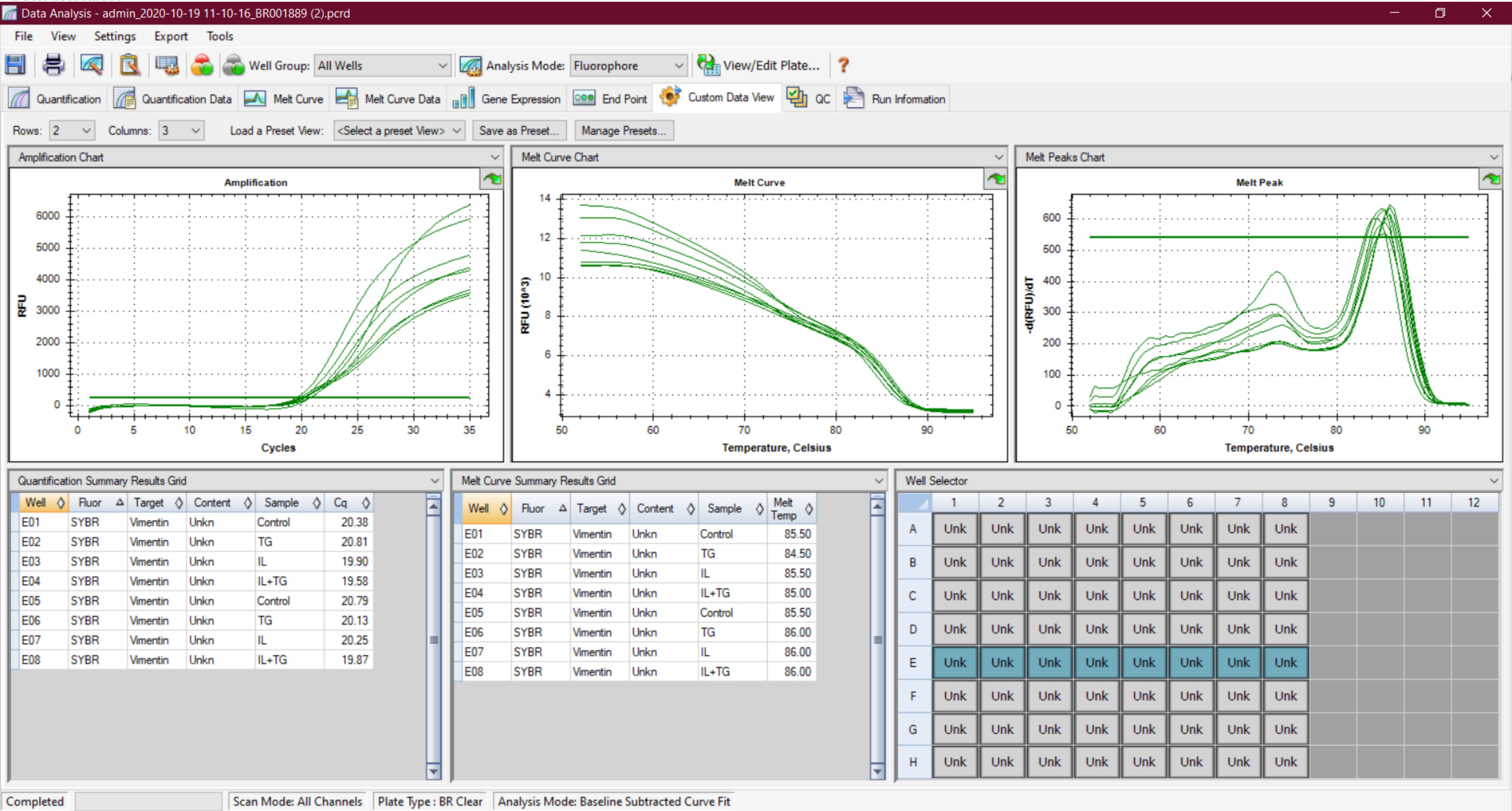

Well	Fluor	Target	Content	Sample	Cq
E01	SYBR	Vimentin	Unkn	Control	20.38
E02	SYBR	Vimentin	Unkn	TG	20.81
E03	SYBR	Vimentin	Unkn	IL	19.90
E04	SYBR	Vimentin	Unkn	IL+TG	19.58
E05	SYBR	Vimentin	Unkn	Control	20.79
E06	SYBR	Vimentin	Unkn	TG	20.13
E07	SYBR	Vimentin	Unkn	IL	20.25
E08	SYBR	Vimentin	Unkn	IL+TG	19.87

Well	Fluor	Target	Content	Sample	Melt Temp
E01	SYBR	Vimentin	Unkn	Control	85.50
E02	SYBR	Vimentin	Unkn	TG	84.50
E03	SYBR	Vimentin	Unkn	IL	85.50
E04	SYBR	Vimentin	Unkn	IL+TG	85.00
E05	SYBR	Vimentin	Unkn	Control	85.50
E06	SYBR	Vimentin	Unkn	TG	86.00
E07	SYBR	Vimentin	Unkn	IL	86.00
E08	SYBR	Vimentin	Unkn	IL+TG	86.00

	1	2	3	4	5	6	7	8	9	10	11	12
A	Unk	Unk	Unk	Unk	Unk	Unk	Unk	Unk				
B	Unk	Unk	Unk	Unk	Unk	Unk	Unk	Unk				
C	Unk	Unk	Unk	Unk	Unk	Unk	Unk	Unk				
D	Unk	Unk	Unk	Unk	Unk	Unk	Unk	Unk				
E	Unk	Unk	Unk	Unk	Unk	Unk	Unk	Unk				
F	Unk	Unk	Unk	Unk	Unk	Unk	Unk	Unk				
G	Unk	Unk	Unk	Unk	Unk	Unk	Unk	Unk				
H	Unk	Unk	Unk	Unk	Unk	Unk	Unk	Unk				

TGFR-mRNA
Date: 19.10.2020

Data Analysis - admin_2020-10-19 11-10-16_BR001889 (2).pcrd

File View Settings Export Tools

Well Group: All Wells | Analysis Mode: Fluorophore | View/Edit Plate...

Quantification | Quantification Data | Melt Curve | Melt Curve Data | Gene Expression | End Point | Custom Data View | QC | Run Information

Rows: 2 Columns: 3 Load a Preset View: <Select a preset View> Save as Preset... Manage Presets...

Amplification Chart

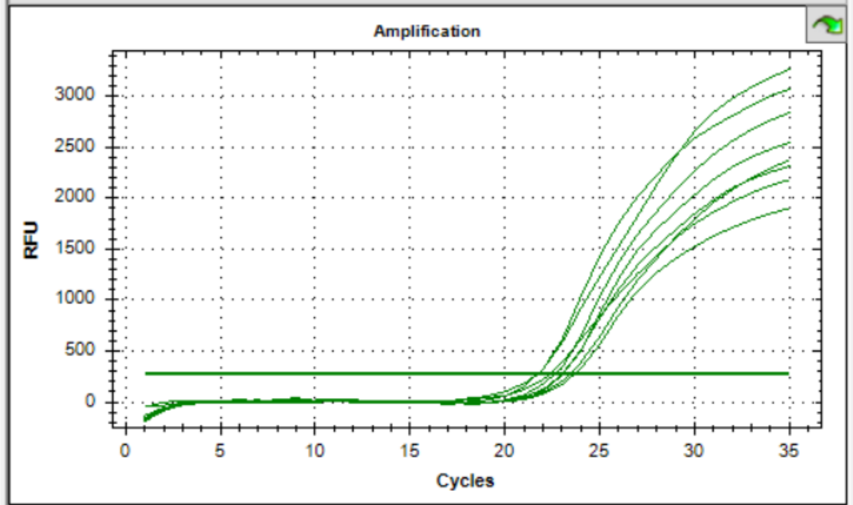

Melt Curve Chart

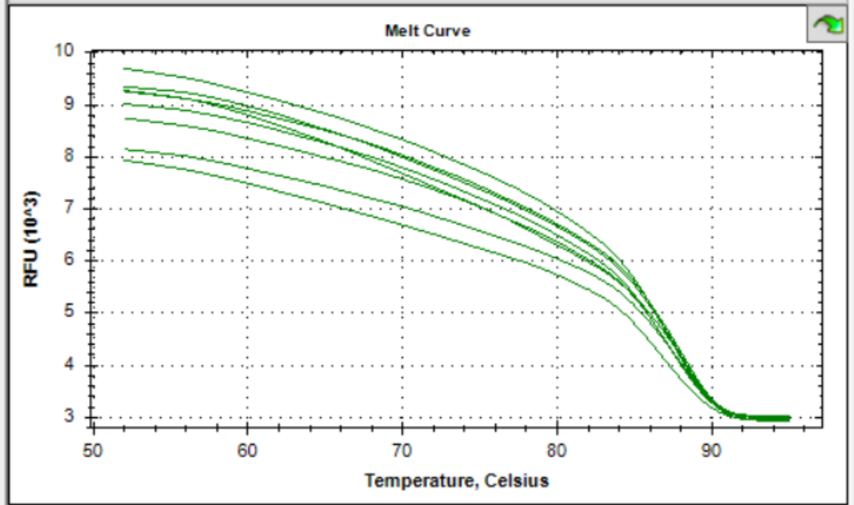

Melt Peaks Chart

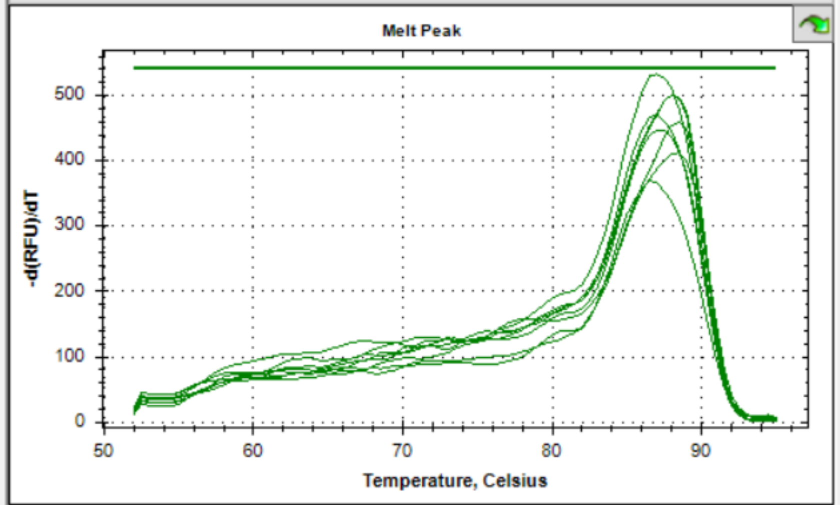

Quantification Summary Results Grid

Well	Fluor	Target	Content	Sample	Cq
H01	SYBR	TGFR	Unkn	Control	21.72
H02	SYBR	TGFR	Unkn	TG	22.67
H03	SYBR	TGFR	Unkn	IL	23.08
H04	SYBR	TGFR	Unkn	IL+TG	21.75
H05	SYBR	TGFR	Unkn	Control	23.04
H06	SYBR	TGFR	Unkn	TG	23.42
H07	SYBR	TGFR	Unkn	IL	23.70
H08	SYBR	TGFR	Unkn	IL+TG	22.37

Melt Curve Summary Results Grid

Well	Fluor	Target	Content	Sample	Melt Temp
H01	SYBR	TGFR	Unkn	Control	None
H02	SYBR	TGFR	Unkn	TG	None
H03	SYBR	TGFR	Unkn	IL	None
H04	SYBR	TGFR	Unkn	IL+TG	None
H05	SYBR	TGFR	Unkn	Control	None
H06	SYBR	TGFR	Unkn	TG	None
H07	SYBR	TGFR	Unkn	IL	None
H08	SYBR	TGFR	Unkn	IL+TG	None

Well Selector

	1	2	3	4	5	6	7	8	9	10	11	12
A	Unk	Unk	Unk	Unk	Unk	Unk	Unk	Unk				
B	Unk	Unk	Unk	Unk	Unk	Unk	Unk	Unk				
C	Unk	Unk	Unk	Unk	Unk	Unk	Unk	Unk				
D	Unk	Unk	Unk	Unk	Unk	Unk	Unk	Unk				
E	Unk	Unk	Unk	Unk	Unk	Unk	Unk	Unk				
F	Unk	Unk	Unk	Unk	Unk	Unk	Unk	Unk				
G	Unk	Unk	Unk	Unk	Unk	Unk	Unk	Unk				
H	Unk	Unk	Unk	Unk	Unk	Unk	Unk	Unk				

Completed | Scan Mode: All Channels | Plate Type : BR Clear | Analysis Mode: Baseline Subtracted Curve Fit

B-Actin-mRNA
Date: 19.10.2020

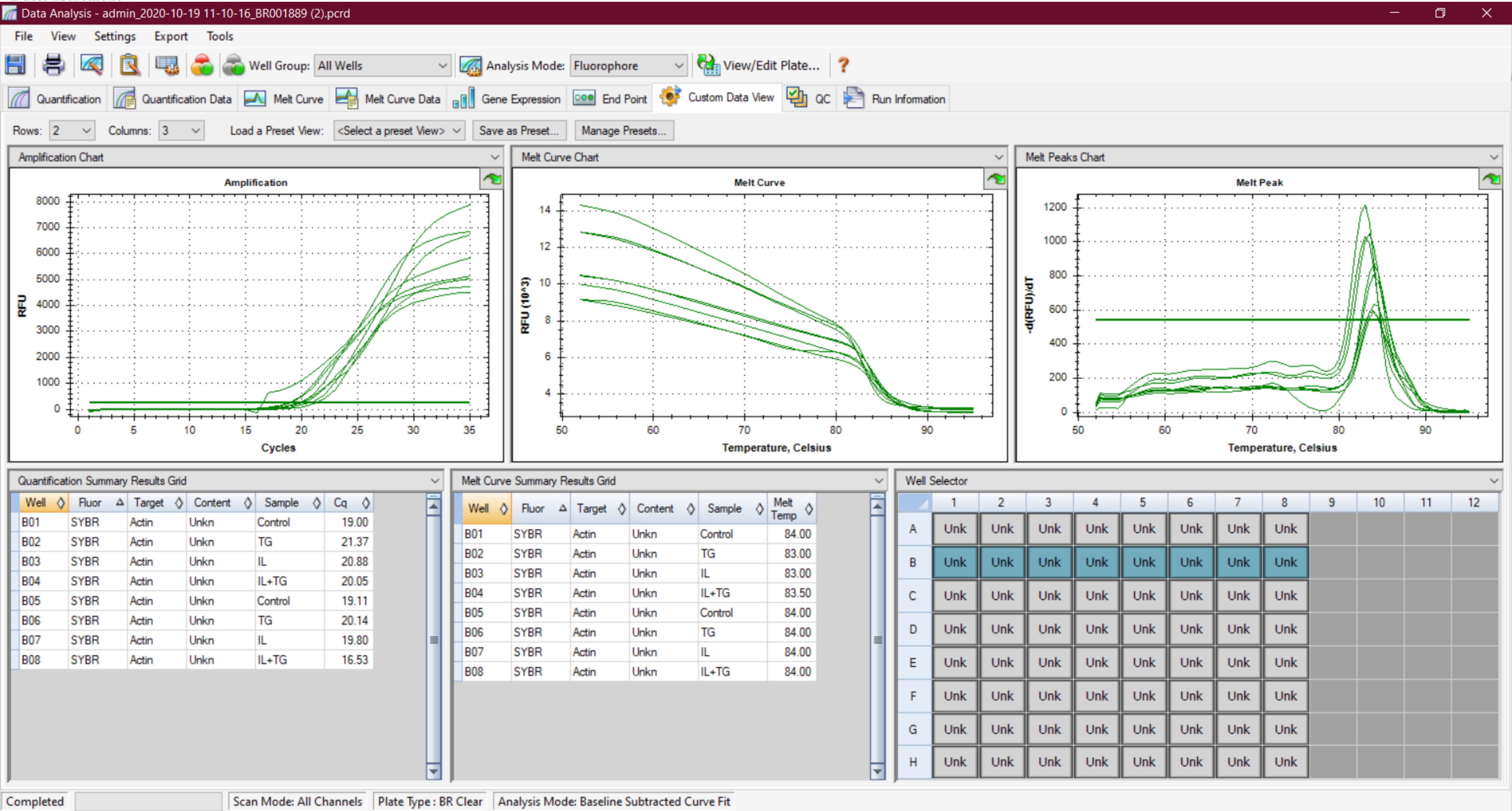

Quantification Summary Results Grid

Well	Fluor	Target	Content	Sample	Cq
B01	SYBR	Actin	Unkn	Control	19.00
B02	SYBR	Actin	Unkn	TG	21.37
B03	SYBR	Actin	Unkn	IL	20.88
B04	SYBR	Actin	Unkn	IL+TG	20.05
B05	SYBR	Actin	Unkn	Control	19.11
B06	SYBR	Actin	Unkn	TG	20.14
B07	SYBR	Actin	Unkn	IL	19.80
B08	SYBR	Actin	Unkn	IL+TG	16.53

Melt Curve Summary Results Grid

Well	Fluor	Target	Content	Sample	Melt Temp
B01	SYBR	Actin	Unkn	Control	84.00
B02	SYBR	Actin	Unkn	TG	83.00
B03	SYBR	Actin	Unkn	IL	83.00
B04	SYBR	Actin	Unkn	IL+TG	83.50
B05	SYBR	Actin	Unkn	Control	84.00
B06	SYBR	Actin	Unkn	TG	84.00
B07	SYBR	Actin	Unkn	IL	84.00
B08	SYBR	Actin	Unkn	IL+TG	84.00

Well Selector

	1	2	3	4	5	6	7	8	9	10	11	12
A	Unk	Unk	Unk	Unk	Unk	Unk	Unk	Unk				
B	Unk	Unk	Unk	Unk	Unk	Unk	Unk	Unk				
C	Unk	Unk	Unk	Unk	Unk	Unk	Unk	Unk				
D	Unk	Unk	Unk	Unk	Unk	Unk	Unk	Unk				
E	Unk	Unk	Unk	Unk	Unk	Unk	Unk	Unk				
F	Unk	Unk	Unk	Unk	Unk	Unk	Unk	Unk				
G	Unk	Unk	Unk	Unk	Unk	Unk	Unk	Unk				
H	Unk	Unk	Unk	Unk	Unk	Unk	Unk	Unk				

Analysis of RT-PCR data

	A	B	C	D	E	F	G
1	23.10.202(	B-Actin		Average			
2	Control	23.01705	22.76345	22.89025	0		
3	TGF	24.52315	23.84369	24.18342	-1.29317		
4	IL6	32.02353	30.93007	31.4768	-8.58655		
5	IL+TG	23.53371	22.52659	23.03015	-0.1399		
6							
7		IL6R					
8	Control	26.28971	26.4026	26.34616	0	0	1
9	TGF	26.44641	26.18687	26.31664	0.029517	2.41764	5.342962
10	IL6	26.27796	26.10805	26.19301	0.153151	8.290549	313.1151
11	IL+TG	26.63457	27.30994	26.97226	-0.6261	0.095647	1.068544
12							
13		gp130					
14	Control	21.61224	21.1439	21.37807	0	0	1
15	TGF	22.39862	21.56415	21.98138	-0.60331	1.784808	3.445726
16	IL6	21.90933	21.64164	21.77549	-0.39742	7.739978	213.7792
17	IL+TG	21.6091	20.68955	21.14933	0.228741	0.950486	1.932524
18							

	A	B	C	D	E	F	G
13							
14	22.10.202(	B-Actin					
15	Control	23.34234	23.34239	23.34237	0		
16	TGF	25.18908	25.61695	25.40302	-2.06065		
17	IL6	31.86427	33.00877	32.43652	-9.09415		
18	IL+TG	24.16495	26.62763	25.39629	-2.05392		
19							
20		PCNA					
21	Control	26.27984	27.34326	26.81155	0	0	1
22	TGF	26.52684	26.15262	26.33973	0.471817	2.532466	5.785599
23	IL6	27.12955	25.94995	26.53975	0.271796	9.365949	659.8292
24	IL+TG	28.00642	26.44302	27.22472	-0.41317	1.640751	3.118281
25							
26		Ki67					
27	Control	28.07971	28.28772	28.18372	0	0	1
28	TGF	28.46172	27.6639	28.06281	0.120908	2.181558	4.53643
29	IL6	29.32152	31.19547	30.2585	-2.07478	7.019375	129.7306
30	IL+TG	28.13156	28.13395	28.13276	0.05096	2.104884	4.301632

19.10.202(	B-Actin					
Control	19.00414012	19.11056	19.05735	0		
TGF	21.37081703	20.14474	20.75778	-1.70043		
IL6	20.87730744	19.79689	20.3371	-1.27975		
IL+TG	20.05448726	16.53382	18.29415	0.763196		
	N-Cad					
Control	26.46	26.62	26.54	0	0	1
TGF	26.24	26.41	26.325	0.215	1.915427	3.772253
IL6	26.26	26.78	26.52	0.02	1.299751	2.461864
IL+TG	25.12	25	25.06	1.48	0.716804	1.643537
	Vimentin					
Control	20.38	20.79	20.585	0	0	1
TGF	20.81	20.13	20.47	0.115	1.815427	3.519637
IL6	19.9	20.25	20.075	0.51	1.789751	3.457552
IL+TG	19.58	19.87	19.725	0.86	0.096804	1.069402
	TGFR					
Control	21.72	23.04	22.38	0	0	1
TGF	22.67	23.42	23.045	-0.665	1.035427	2.04972
IL6	23.08	23.7	23.39	-1.01	0.269751	1.2056
IL+TG	21.75	22.37	22.06	0.32	-0.4432	0.735503
